# Characterising investments in EU fisheries and defining their desirability

**DOI:** 10.1016/j.fishres.2019.105396

**Published:** 2020-01

**Authors:** Natacha Carvalho, John Casey, Jordi Guillen, Philip Rodgers

**Affiliations:** a European Commission, Joint Research Centre (JRC), Via E. Fermi 2748, Ispra, 21027, Italy; b Lincoln International Business School, University of Lincoln, Lincoln, United Kingdom

**Keywords:** Capital, Overcapacity, Overcapitalisation, Net value added (NVA), Maximum economic yield (MEY)

## Abstract

In most economic sectors, increases in capital (i.e., investments) are often considered virtuous, indicating confidence in the future and expected growth. In fisheries, however, investments are often harmful, as they may lead to increases in fleet capacity, which is not desirable considering the fully exploited or overexploited status of most fish stocks (natural capital), and the dissipation of the resource rent (overcapitalisation). In the EU, the number of fishing vessels have been decreasing for many years, but the fishing capacity is often claimed to have increased. In other words, there are less vessels, but the remaining ones have a higher fishing capacity. In this study, we analyse the evolution of the EU fishing industry’s investments for the period 2008–2016, and whether these investments have been beneficial. Results show that despite the overall decrease in the number of vessels and their average value, investments in some fleets have increased. Moreover, investment decisions (i.e., whether to invest or disinvest) have been more accurate in recent years, leading to a better economic performance. However, results vary by the scale of the fishing activity (small-scale and large scale fleets) and sea basin (Northeast Atlantic Ocean and Mediterranean Sea).

## Highlights

• Between 2008 and 2016, the fleet decreased in number of vessels and in capital value.
• Vessel value has been relatively constant for the period analysed.
• NVA was positive for more than 80% of the fleets analysed and increasing.
• The right investment decision took place in about 60% of the cases between 2014 and 2016.

## 1 Introduction

The possibility to increase individual profits by increasing inputs beyond the level required to achieve the maximum economic yield (MEY), leads to the existence of overcapacity in poorly-managed fisheries, and consequently of overcapitalisation, depletion of the fish stocks and dissipation of the resource rent (Clark, 1990; Pauly et al., 2002; Berkes et al., 2006; Willman et al., 2009; Worm et al., 2009). Managing fisheries worldwide at MEY or at maximum sustainable yield (MSY) would require drastic reductions in existing fishing capacity (Clark, 1990; Pauly et al., 2002; Willman et al., 2009; Sumaila et al., 2012; Merino et al., 2014; Guillen et al., 2016). Indeed, Willman et al. (2009) estimated that an extra $50 billion in rents could be generated annually if global fishing capacity were cut by half. However, although reductions in fishing capacity in terms of numbers of vessels have occurred in many highly developed countries’ fisheries (Bell et al., 2017), increased investment and development of improved fishing technologies have often resulted in increased catchability^2^ . Hence, reductions in the numbers of vessels participating in a fishery may not lead to desired reductions in exploitation rates (fishing mortality).

To understand the extent of the problems associated with overcapacity and overcapitalisation in fisheries, requires an understanding of the relationship between capital, investment and realised exploitation rates. According to Ackley (1978), it is important to differentiate between the theory of capital and the theory of investment, even though they are closely related. The theory of capital deals with variation in capital levels and addresses the question of what is the optimal amount of a particular type of capital. Conversely, the theory of investment is about flows; i.e., what is the optimal rate at which capital should vary when the amount of capital is not optimal. In this sense, investment is defined as the change over time in the amount of capital. Clark and Munro (1975) were the first to connect the theory of capital and the theory of investment in fisheries. Subsequently the link between capital and investment theories in the fisheries sector has been further reviewed, (see for instance, Greboval and Munro, 1999; Charles, 2007).

In fisheries, as for other renewable resources, four different types of capital can be identified: natural, physical, human, and immaterial capital. Natural capital refers to the fish stocks (the natural resources under consideration), which are accessible to fishing firms (Scott, 1955). Physical capital relates to the fishing vessels (the production factor where production capital is accumulated); but a more comprehensive definition would include all the equipment and infrastructure associated with fishing activities. Human capital indicates the labour inputs needed to catch fish. While, the immaterial capital relates to the capital required for the intangible assets; for example, the requirement to own fishing rights to operate in rights-regulated fisheries.

Most studies have focused on the relationship between natural and physical capital; thereby addressing a dual investment problem, where investment decisions can be taken with regards to the natural resource and the physical capital required to harvest the resource (Clark et al., 1979)^3^ . Investment in natural capital (i.e., fish stocks) usually implies determining an exploitation rate (i.e., catches), which would be expected to result in a desired level of unexploited fish stock biomass.

Investment in the physical capital (e.g. number and size of vessels) usually implies determining the fishing capacity needed to achieve a certain exploitation rate. Early works by Smith (1968, 1969) represented from a theoretical perspective, how decisions to invest (enter or exit) in a fishery depend on anticipated profitability; the anticipated levels of returns net of opportunity costs. Results from several empirical studies on the factors that may influence investment in specific fisheries indicate that participation in a fishery depends on expected future revenues, stock status of main target species, first-sale fish prices, operating costs (e.g. fuel), total fleet size (a proxy for congestion), the opportunity cost of capital (i.e., profitability of alternative fisheries), the impact of management measures (e.g. total allowable catches), capital tax costs and depreciation policies, vessel age and vessel size (Clark and Lamberson, 1982; Bockstael and Opaluch, 1983; Opaluch and Bockstael, 1984; Ward and Sutinen, 1994; Jensen, 1998; Mardle et al., 2005; Tidd et al., 2011).

Following Smith and Hanna (1990), fishing capacity can be disaggregated into four components: (i) number of vessels; (ii) size of the vessels; (iii) technical efficiency of vessel operation; and (iv) potential fishing time of each vessel, per specified period of time, e.g. year or season. Thus, physical capital investments in fishing capacity can be accomplished by: (i) increasing the number of vessels; (ii) replacing less-efficient vessels with vessels that are more-efficient; and (iii) improving the technical efficiency of existing vessels (e.g. by incorporating improved fishing gear, more powerful engines or fish detection equipment). Investments in physical capital typically result in increases in catchability with the aim to increase catches, which often leads to a decrease in the natural capital.

Research on capital dynamics has traditionally assumed physical capital to be perfectly malleable, and consequently capital behaves similar to variable costs (Gordon, 1954; Schaefer, 1954; Smith, 1968, 1969; Burt and Cummings, 1970). This means that in fisheries, investments and disinvestments in physical capital can take place (e.g. free entry and exit of vessels) to adjust the fishing capacity to the desired exploitation rate of the natural capital.

However, often exists constraints on investments (and disinvestments) in capital assets, which mean that to some extent, physical capital is non-malleable (Arrow, 1968; Arrow and Kurz, 1970). In fisheries, non-malleability or imperfect malleability of capital occurs because capital cannot easily be shifted in or out of the fishery, thereby entailing a certain cost (Clark et al., 1979; Clark, 1980; Ward and Sutinen, 1994; Munro, 2010). For example, in many fisheries managed using input controls, measures are often in place to prevent additional investment, such as a closed list of licences (Pascoe et al., 2017). However, to date, most of the literature has focused on the decisions to enter or exit fisheries. In fact, vessels are more likely to enter a fishery when profits increase than they are to leave the fishery when profits decline. This is especially true if no profitable alternative fisheries are available, because fishers have paid a high entry fixed (sunk) cost, equal to the vessel value (Dixit, 1989; Ward and Sutinen, 1994; Ikiara and Odink, 1999). In addition, high switching costs when changing fisheries tend to limit capacity reallocation and fishers tend to stay within the same fishery over time (Bockstael and Opaluch, 1983; Opaluch and Bockstael, 1984; Quillérou et al., 2013)^4^ . Nevertheless, the degree of (non-)malleability of physical capital varies significantly from fishery to fishery.

Therefore, imperfect malleability of physical capital may justify a degree of overcapitalisation in a fishery (Clark et al., 1979). Thus, even if the optimal long-run equilibrium is unaffected by the degree of malleability of capital, the short-run optimal strategies can be significantly affected. Consequently, the general theory of capital is often not applicable in fisheries bio-economics.

Few empirical studies^5^ investigate investment in fisheries, its behaviour and drivers (Kirkley and Squires, 1988; Kirkley et al., 2002; Nøstbakken et al., 2011). Possible explanations for the paucity of such studies, are the limited data available on investment and capital in marine fisheries (Kirkley and Squires, 1988) and the absence of a unique and agreed measurement of capacity (Kirkley et al., 2002).

Economic and biological data for the EU fishing fleet, including capital and investment, have been systematically collected under the Data Collection Framework (DCF) since 2008 (European Union, 2008; STECF, 2017). According to STECF (2017), the capacity of the EU fleet decreased gradually between 2008 and 2016: the number of vessels decreased 13%, horse power by 15% and gross tonnage by 19%.

We are interested to know how investments in the EU fleet fishing fleet physical capital have been taking place. Investments and disinvestments (i.e., changes in the value of the fleet) can be accommodated by changing the number of vessels or the value of the vessels in a fleet. Hence, this study uses DCF data to investigate the investment decisions of the EU fishing fleets during the period 2008–2016. This is done by analysing whether the capital value in a fleet has increased or decreased and if that has happened due to changes in the number of vessels or the value of the vessels. More than that, it is analysed whether these investment decisions have delivered positive outcomes for society in terms of added value increases, and so if these investment decisions have been efficient and desirable.

## 2 Materials and methods

### 2.1 Methodology

To determine whether changes in the value of a fleet are economically beneficial, information regarding the current performance of the fleet is required. Such information can be obtained from observed trends in profitability. We propose to use Net Value Added (NVA), the sum of the returns to both capital (i.e., net profit) and labour (i.e., salaries), as a measure of profitability as it is generally regarded as a better means of defining the returns to society than using profits alone (Chen et al., 2005; Guillen et al., 2015).

Net value added is the value of output less the values of both intermediate consumption and consumption of fixed capital. Hence, we estimate NVA as:

(1) NVA=Total Revenues – Fuel costs – Other variable costs – Repair and maintenance costs - Other non-variable costs – Depreciation costs

The value of the fleet (i.e., capital) is estimated as the tangible assets value, measured as the depreciated replacement value, as collected under the EU data collection framework (DCF). The variation of capital from year t-1 to year t can be considered as the real investment in capital (capital flow) taking place in a fleet.

Based on the above observations we can identify the basis for a decision rule to determine whether investments are, or have been, effective from a society’s welfare point of view:

• When investments or disinvestments lead to increases in the NVA, they can be regarded as effective, heralding improved and sustainable long-run profitability of the fishery;
• When investments or disinvestments lead to decreases in the NVA, they can be regarded as detrimental to the long-run profitability of the fishery.

The outcomes of investments can be determined for different stratifications, e.g. fishing region, country, and fleet. We can identify at the fleet level where over- or under-investment occurred.

We consider that investment and disinvestments can be considered effective (i.e., desirable) if they deliver a benefit to society, expressed as an increase in NVA. Using such an approach, we classify the outcome of an investment as one of four types (see Table 1).

• Type 1: investment led to an increase in NVA: Investment was effective.
• Type 2: disinvestment led to an increasing NVA: Disinvestment was effective.
• Type 3: investment led to decreases in NVA: Investment was ineffective; an undesirable situation, urged to disinvest.
• Type 4: disinvestment led to a decrease in NVA: Disinvestment was ineffective or not sufficiently effective. Further disinvestment may have been needed. The resource may have been largely overexploited or economic conditions worsened. It is an undesirable situation.

**Table 1:** Classification of the investments according to the investment effectiveness as a function of changes in the NVA and capital invested.

NVA	Increase	Type 1	Type 2
	Decrease	Type 3	Type 4
		Investment	Disinvestment
		Capital	

### 2.2 Data

The data on the EU fishing fleet used in this study have been assembled from the 2018 Annual Economic Report of the EU fishing fleet (AER; STECF, 2017). The AER uses data collected under the DCF (European Union, 2008) and reported by EU Member States in response to the 2018 fleet economics data call. The data requested were for the years 2008 to 2016.

The AER reported separately by fleet segment^6^ and at overall Member State level the following variables: transversal variables (capacity, landings and effort); economic variables (income, costs, employment, enterprises, capital value and investment). Monetary variables reported as nominal values in the AER were converted to real values, adjusting them by the real inflation rate, following the methodology described in the AER (see for instance, STECF, 2017).

In this study, we analyse data at fleet segment level only for those fleets that have reported all relevant variables for the period 2008–2016. This concerns data for 242 fleets which on average for the period 2008–2016, represented a total of 34,039 vessels (52% of the active EU fleet), €3.87 billion in physical capital (78% of the active fleet), and generated €1.99 billion in NVA (72% of the total). No fleets from France, Greece and Croatia were considered in the analysis because of missing data (for more details, see STECF, 2017). The 242 EU fishing fleets analysed and their average number of vessels, capital value, capital per vessel and net value added for the period 2008–2016 are presented in Supplementary materials 1.

## 3 Results

Information on investment decisions and their outcome by fleet number and weighted by the number of vessels in each fleet are summarised in Tables 2a,b. This analysis has been replicated by sea basin (Northeast Atlantic Ocean and Mediterranean Sea) and fishing activity (small-scale and large scale fleets) and reported in Supplementary materials 2 due to space limitations in the main text.

**Table 2:** Evolution of annual between-year investment and their outputs (changes in NVA) for the EU fleet. Values represent the number of fleets (a) and the number of vessels in each fleet (b) exhibiting the different types of investment behaviour between the year indicated and the preceding year.

a)											
Capital	Number of vessels	Average value per vessel	NVA	2009	2010	2011	2012	2013	2014	2015	2016
Increase	Increase	Increase	Increase	20	23	9	15	20	28	22	29
Increase	Increase	Increase	Decrease	26	15	9	8	10	18	9	5
Increase	Stable	Increase	Increase	5	5	8	9	7	9	13	8
Increase	Stable	Increase	Decrease	7	4	8	7	9	7	9	4
Increase	Increase	Decrease	Increase	12	13	12	15	2	10	13	11
Increase	Increase	Decrease	Decrease	13	14	7	13	10	8	6	3
Increase	Decrease	Increase	Increase	10	19	6	16	8	18	19	20
Increase	Decrease	Increase	Decrease	28	14	14	16	13	14	18	13
Decrease	Decrease	Increase	Increase	10	20	13	10	12	11	15	14
Decrease	Decrease	Increase	Decrease	12	14	14	16	17	9	12	11
Decrease	Increase	Decrease	Increase	11	9	17	12	13	18	14	19
Decrease	Increase	Decrease	Decrease	9	9	12	6	20	6	8	12
Decrease	Stable	Decrease	Increase	9	11	12	12	13	16	14	9
Decrease	Stable	Decrease	Decrease	14	10	7	15	18	12	10	10
Decrease	Decrease	Decrease	Increase	27	27	48	35	26	30	40	46
Decrease	Decrease	Decrease	Decrease	29	35	46	37	44	28	20	28

b)											
Capital	Number of vessels	Average value per vessel	NVA	2009	2010	2011	2012	2013	2014	2015	2016
Increase	Increase	Increase	Increase	3,611	2,149	410	700	1,421	1,250	2,436	3,291
Increase	Increase	Increase	Decrease	957	9,598	5,665	789	550	2,900	1,752	130
Increase	Stable	Increase	Increase	72	131	745	154	177	94	308	186
Increase	Stable	Increase	Decrease	159	749	191	115	336	93	856	114
Increase	Increase	Decrease	Increase	1,223	1,465	2,357	1,393	56	395	928	633
Increase	Increase	Decrease	Decrease	608	867	149	2,775	1,422	1,781	824	176
Increase	Decrease	Increase	Increase	8,854	2,124	911	1,499	936	1,953	5,224	2,416
Increase	Decrease	Increase	Decrease	4,568	3,934	463	2,536	5,418	397	2,278	569
Decrease	Decrease	Increase	Increase	373	2,113	541	524	422	4,544	6,248	9,284
Decrease	Decrease	Increase	Decrease	1,631	740	1,138	2,280	2,712	927	2,201	380
Decrease	Increase	Decrease	Increase	1,264	1,970	4,786	2,896	884	7,623	2,297	5,806
Decrease	Increase	Decrease	Decrease	2,069	2,629	1,704	251	6,916	1,608	920	1,021
Decrease	Stable	Decrease	Increase	192	292	193	359	304	222	523	137
Decrease	Stable	Decrease	Decrease	677	433	183	252	359	712	263	194
Decrease	Decrease	Decrease	Increase	4,094	1,719	6,548	3,741	6,115	4,961	4,094	5,322
Decrease	Decrease	Decrease	Decrease	5,463	4,572	8,170	13,571	5,228	3,620	1,430	2,477

**Fig. 1:**
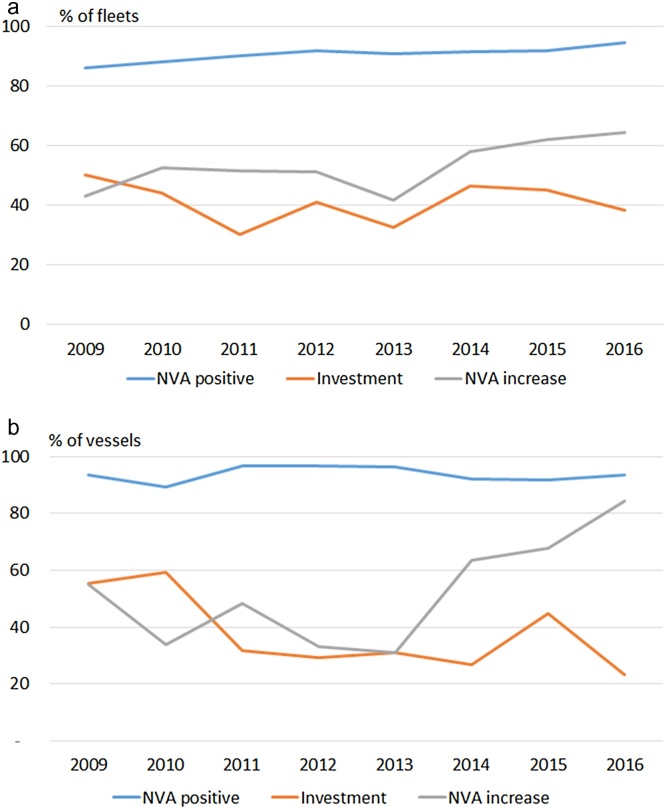
(a, b): Share of fleets (a) and share of vessels by fleet (b) with increasing capital, with increasing NVA and showing positive NVA. Values represent the change in the proportion of exhibiting the different types of investment behaviour between the year indicated and the preceding year, e.g. the value for 2009 represents the change compared to 2008.

During the analysed period, more fleets (of the selected fleets) disinvested than invested. Disinvestment are mostly due to decreases in the number of vessels. When investing, most of the capital increases in the fleet are due to increases in the average value per vessel. Investment expressed as increases in the average value of the vessels (on average in more than 50% of the cases for the period 2008-16) is more common than investment expressed as an increase in the number of vessels (less than 20%); while increases in both the average value of the vessels and in the number of vessels took place in about 30% of the cases (Tables 2a,b). Over the entire period 2008–2016, the total capital investment increased for about 40% of the fleets included in the analysis, and so disinvestment occurred in about 60% of them (Fig. 1a).

The NVA had been positive for more than 85% of the fleets analysed and overall this proportion has gradually increased to almost 95% in 2016. Initially, about 40% of the fleets showed an increase in NVA, and so the NVA decreased for 60% of them. But the proportion of fleets with an increasing NVA increased to about 60% in the last years (Fig. 1a). When weighting this analysis by the number of vessels in each fleet, NVA was positive for more than 90% of the vessels, the proportion of vessels with an increasing NVA increased to more than 80%, while the proportion of vessels investing decreased from almost 60% to slightly more than 20% (Fig. 1b). These differences in the results between the proportion of fleets and the proportion of vessels are because small-scale fleets are on average compounded by a larger number of vessels than large-scale fleets.

**Fig. 2:**
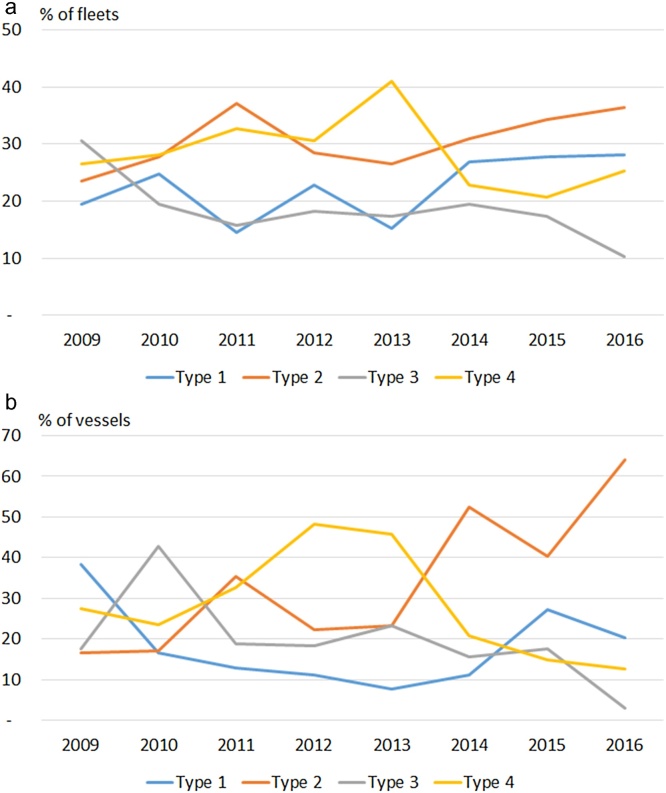
(a, b): Evolution of investment types (in %) for the EU fleets analysed between 2008 and 2016. Values represent the change in the proportion of fleets (a) and vessels by fleet (b) exhibiting the different types of investment behaviour between the year indicated and the preceding year.

When this analysis is replicated by sea basin and fishing activity, it can be seen that Northeast Atlantic fleets perform on average better and more fleets are investing than Mediterranean fleets. Moreover, large-scale fleets outperform the small-scale fleets, in particular in the Mediterranean Sea (see Supplementary materials 2) (Fig. 2).

Next, it is investigated the desirability of investment decisions in the EU fishing fleets, i.e., when these investment decisions led to increases in the NVA.

Of the 242 fleets examined for 2016, 6 fleets (29%) showed Type 1 outcomes of investment (investment is efficient since increases in investment lead to increases in added value). While, 88 fleets (36%) showed Type 2 investment outcomes (disinvestment is effective as it leads to increases in NVA), 25 fleets (10%) showed Type 3 investment outcomes (investment was ineffective; an undesirable situation that stresses the need to disinvest as increases in investment lead to decrease in added value) and 61fleets (25%) showed Type 4 investment outcomes (disinvestment ineffective or insufficiently effective: further disinvestment may be needed as value added is still decreasing).

When looking at the distribution of investment types considering the vessels in each fleet, resulted that 64% of the fleets showed Type 2 investment outcomes, 20% showed Type 1, 13% showed Type 4, and 3% showed Type 3 investment outcomes. This shows that a large number of small-scale fleets reached increases in NVA by decreasing capital, i.e., disinvesting (see also Supplementary material 2).

When an investment in a fleet occurred, it increasingly led to increases in the NVA (Type 1/(Type 1+Type 3)), from less than 40% in 2009 to more than 70% of the cases in 2016. While, when a disinvestment in a fleet occurred, it led to increases in the NVA (Type 2/(Type 2+Type 4)) from less than 50% in 2009 to about 60% of the cases in 2016.

## 4 Discussion

Between 2008 and 2016, the EU fleet decreased similarly in number of vessels and in capital value, the average value per vessel remaining relatively similar between 2008 and 2016 (own calculations from STECF, 2017 data). This would signal a reduction of the fishing capacity, expected as the value per vessel has been relatively constant, but the number of vessels has decreased.

Of those fleets where investment took place, increases in the average value of vessels occurred in most cases, while disinvestments are mostly due to decreases in the number of vessels.

A major difficulty for fisheries managers, is to determine the optimal level of fleet capacity because the optimal level will change overtime depending on the status of the resources the fleet exploits and the available fishing opportunities. Consequently, if fleets are to take advantage of varying fishing opportunities, the optimum fleet capacity will at certain points in time, include spare or underutilised capacity. Similarly, industry’s investment decisions are inextricably linked with economic and biological uncertainty (e.g. Arrow and Fisher, 1974; Brennan and Schwartz, 1985). In an attempt to provide an objective means to assess the desirability of investment in fisheries we propose a 4-stage classification of investment outputs.

Based on our proposed classification of investment outcomes (Types 1–4) we observe that:

• During the period 2009–2016, Type 1 desirability of investment peaked in recent years to almost 30%, while in initial periods it oscillated between 15% and 25%. Type 2 peaked in 2011, falling to almost 25% in 2012 and 2013, but increasing since then up to similar values than those in 2011.
• In 2016, 28% of the fleets analysed showed Type 1, 36% showed Type 2, 10% showed Type 3, and 25% showed Type 4.

We further propose that only Type 1 and Type 2 can be considered satisfactory. Therefore, the right investment decision, from a social point of view, took place in about 60% of the disinvestments case and more than 70% of investment cases in recent years. While, NVA was positive for more than 80% of the fleets analysed, increasing up to 90% in recent years.

Thus, results show that despite the overall decrease in the number of vessels, the EU fishing fleet has been able to invest and disinvest in the right fleets, especially in recent years. However, results vary slightly by sea basin and fishing activity, with the exception of small-scale Mediterranean fleets, which perform worse than the rest. This can be explained by fish stocks in the Mediterranean not being restored, which lead to low economic performance, and therefore, fishers are less keen to invest (D-Rocha et al., 2019).

Hence, in the authors’ knowledge, this is the first time that investment decisions are analysed for such a large number of vessels and fleets; the few empirical studies that investigate investment in fisheries are often at the fishery level (e.g. Clark and Lamberson, 1982; Bockstael and Opaluch, 1983; Opaluch and Bockstael, 1984; McKelvey, 1987; Bjørndal and Conrad, 1987; Ward and Sutinen, 1994; Jensen, 1998; Mardle et al., 2005; Tidd et al., 2011).

Several limitations must be taken into account when interpreting the decision rule set out above. Firstly, fisheries are dynamic: fish stocks, labour, demand and costs are constantly subject to change. Consequently, the optimal economic position in terms of NVA_max_^7^ also changes constantly. The net effect of such changes is that the fisheries tend towards the equilibrium position (i.e., open access) while managers often target MSY or MEY, rather than achieving an absolutely stable position. Such apparent contradictions nevertheless do not detract from the utility of our proposed investment classification because the changes in investments and in NVA are captured in the classification of investment types. However, classifying investments in this way does not provide any reliable predictive power, because what could be optimal one year, may not be optimal in subsequent years. This also explains part of the inter-year oscillations.

The bio-economic model, and so the decision rule, relies on a deterministic model of the fish stocks which assumes that changes occur only as a result of the physical ability of the fish to reproduce and grow, a constant rate of natural mortality and as a result of fishing. Fish stocks are, however, subject to additional natural variability that cannot be modelled and therefore it adds variability and uncertainty to the system. The analysis assumes a static long run equilibrium; however, in the short run, the optimal reference points may imply a higher level of exploitation because of the existence of a social time preference discount rate (Pontecorvo and Schrank, 2009). In any case, similar conclusions may be drawn from both analyses.

There may be a time lag between the time that the decision to invest takes place, the timing of the investment spend and the time that any resulting costs and benefits originated from this investment occur. For example, Bjørndal and Conrad (1987), analysing the North Sea herring fishery, estimated that when profits are positive, new vessels would take two years to join the fishery (the time required to build a new purse seine vessel). For the fleets in our study, such a time lag effect may be vague since vessels may be allocated to different fleets in different years depending on the main fishing activity (predominant fishing technique), as well as the availability of inactive vessels. In this sense, inactive vessels behave as latent capacity. In 2015, there were 20 444 inactive vessels, about 24% of the whole EU fleet. The existence of a significant amount of inactive vessels adds some flexibility to the system; i.e., re-activation of inactive vessels under more favourable conditions (e.g. increased fishing opportunities) would represent investment. Conversely, under less favourable conditions, vessels can become inactive and cease to operate, in which case, they are not considered in our analysis, but they would have to bear capital costs. However, the existence of inactive vessels implies the existence of capital costs for those vessels, in addition to other potential costs such as mooring or maintenance. Expectations on capacity adjusting subsidies (i.e., buy-back programs) could lead to the existence of a higher level of inactive vessels.

The data in our analysis relate to vessels aggregated at the level of the fleet and are assumed to show the evolution of investment decisions of a group of vessels with similar fishing activity. In reality, however, within a fleet, the performance by individual vessels can be quite different. Unfortunately, sufficient data at the vessel level are not available to examine inter-vessel investment decisions.

## Footnotes

2 Catchability (q) is a concept that reflects the efficiency of a particular fishery. It relates the fishing mortality rate (F) generated on a fish stock by a fishery to the deployed fishing effort (E): F = q * E. An increase in catchability in a fishery equates to an increase in catching efficiency.

3 It should be noted that individual fishing firms can normally decide on the physical, human, and immaterial capital levels, through investing and disinvesting; but rarely on the natural capital. This is because as individual firms, they do not have a tangible means to invest in fish stocks. Decisions regarding the natural capital level and consequently on iinvestment decisions, can only be taken by managers or sole-owners of a fishery.

4 In many fisheries worldwide, physical capital investment decisions are also driven by the non-malleability of human capital, which includes the relatively low risk of losing the fishing job, low overhead costs, and the possibility of massive losses (following exit) for those with large investments in the fishing sector, due to low second-hand prices for fishing equipment (Oduor-Otieno et al., 1978; Ikiara and Odink, 1999).

5 See for instance, Clark and Lamberson, 1982; Bockstael and Opaluch, 1983; Opaluch and Bockstael, 1984; McKelvey, 1987; Bjørndal and Conrad, 1987; Ward and Sutinen, 1994; Jensen, 1998; Mardle et al., 2005; Tidd et al., 2011.

6 A fleet segment is the combination of a particular fishing technique category and a vessel length category (see STECF, 2017).

7 Conversely, MSY is not dependent on economic factors, but on changes on the stock dynamics, fishing patterns and environmental conditions.
