# Supplementary material for: Characterising investments in EU fisheries and defining their desirability
Source: Fish Res. 2020 Jan;221:105396. doi: 10.1016/j.fishres.2019.105396 (PMC6853412; doi:10.1016/j.fishres.2019.105396)
Supplement: Supplementary file 1 [file mmc1.docx]

**Supplementary material 1**

Table S.M.1: Average number of vessels, capital value, capital per vessel and net value added for the 242 EU fishing fleets analysed for the period 2008-2016

| Fleet name | Country | Sea basin | Islands | Scale | Number of vessels | Capital value | Capital per vessel | Net Value Added |
| --- | --- | --- | --- | --- | --- | --- | --- | --- |
| BGR MBS DFN0006 NGI | BGR | MBS |  | SCF | 264 | 707,234 | 2,709 | 86,897 |
| BGR MBS DFN0612 NGI | BGR | MBS |  | SCF | 397 | 2,206,222 | 5,603 | 718,238 |
| BGR MBS FPO0006 NGI | BGR | MBS |  | SCF | 6 | 13,417 | 2,312 | 7,675 |
| BGR MBS FPO0612 NGI | BGR | MBS |  | SCF | 35 | 207,733 | 6,094 | 42,295 |
| BGR MBS HOK0006 NGI | BGR | MBS |  | SCF | 22 | 54,985 | 2,566 | - 6,389 |
| BGR MBS HOK0612 NGI | BGR | MBS |  | SCF | 44 | 222,003 | 5,116 | 57,259 |
| BGR MBS PGP0612 NGI | BGR | MBS |  | SCF | 17 | 237,695 | 12,392 | -18,126 |
| BGR MBS PMP0006 NGI | BGR | MBS |  | SCF | 33 | 70,898 | 2,118 | 49,195 |
| BGR MBS PMP0612 NGI | BGR | MBS |  | SCF | 125 | 868,449 | 6,985 | 647,114 |
| CYP MBS PG 0006 NGI | CYP | MBS |  | SCF | 36 | 1,017,940 | 27,276 | -14,298 |
| CYP MBS PG 0612 NGI | CYP | MBS |  | SCF | 412 | 27,493,682 | 66,112 | -2,066,087 |
| ESP MBS HOK0612 NGI | ESP | MBS |  | SCF | 90 | 1,365,337 | 14,714 | 3,686,235 |
| ITA MBS PGP0006 NGI | ITA | MBS |  | SCF | 2,367 | 16,386,628 | 6,914 | 36,400,978 |
| ITA MBS PGP0612 NGI | ITA | MBS |  | SCF | 5,307 | 125,559,359 | 23,646 | 96,644,994 |
| MLT MBS HOK0006 NGI | MLT | MBS |  | SCF | 25 | 302,919 | 11,073 | -44,861 |
| MLT MBS HOK0612 NGI | MLT | MBS |  | SCF | 70 | 4,069,224 | 62,070 | 94,832 |
| MLT MBS PGP0006 NGI | MLT | MBS |  | SCF | 297 | 3,169,195 | 10,719 | -81,955 |
| MLT MBS PGP0612 NGI | MLT | MBS |  | SCF | 161 | 5,141,333 | 31,593 | -287,416 |
| MLT MBS PMP0612 NGI | MLT | MBS |  | SCF | 90 | 3,473,590 | 45,579 | 26,153 |
| ROU MBS PG 0612 NGI | ROU | MBS |  | SCF | 149 | 820,666 | 5,989 | 545,234 |
| SVN MBS DFN0006 NGI | SVN | MBS |  | SCF | 31 | 124,436 | 3,984 | 145,984 |
| SVN MBS DFN0612 NGI | SVN | MBS |  | SCF | 37 | 1,214,400 | 32,560 | 586,270 |
| POL NAO PG 0010 | POL | NAO |  | SCF | 475 | 19,044,633 | 40,165 | 4,763,782 |
| POL NAO PG 1012 | POL | NAO |  | SCF | 84 | 10,238,376 | 122,836 | 1,316,049 |
| DEU NAO PG 0010 NGI | DEU | NAO |  | SCF | 793 | 5,195,322 | 6,535 | 1,310,379 |
| DEU NAO PG 1012 NGI | DEU | NAO |  | SCF | 68 | 2,165,841 | 31,770 | 473,106 |
| DNK NAO PGP0010 NGI | DNK | NAO |  | SCF | 901 | 21,423,600 | 24,038 | 5,187,450 |
| DNK NAO PGP1012 NGI | DNK | NAO |  | SCF | 57 | 5,874,011 | 103,375 | 1,982,108 |
| ESP NAO HOK1012 NGI | ESP | NAO |  | SCF | 68 | 1,364,083 | 20,172 | 2,525,533 |
| EST NAO PG 0010 NGI | EST | NAO |  | SCF | 1,046 | 4,688,758 | 4,751 | 1,468,912 |
| EST NAO PG 1012 NGI | EST | NAO |  | SCF | 85 | 2,828,819 | 33,044 | 772,251 |
| FIN NAO PG 0010 NGI | FIN | NAO |  | SCF | 1,548 | 18,902,157 | 12,291 | -156,167 |
| FIN NAO PG 1012 NGI | FIN | NAO |  | SCF | 54 | 2,996,131 | 55,989 | -425,161 |
| GBR NAO DFN0010 NGI | GBR | NAO |  | SCF | 640 | 13,149,570 | 20,618 | 6,990,570 |
| GBR NAO DFN1012 NGI | GBR | NAO |  | SCF | 24 | 5,014,364 | 210,584 | 2,842,614 |
| GBR NAO FPO0010 NGI | GBR | NAO |  | SCF | 1,808 | 46,260,858 | 25,658 | 32,578,613 |
| GBR NAO FPO1012 NGI | GBR | NAO |  | SCF | 178 | 11,373,696 | 64,028 | 11,070,172 |
| GBR NAO HOK0010 NGI | GBR | NAO |  | SCF | 493 | 7,838,077 | 16,102 | 3,095,007 |
| GBR NAO HOK1012 NGI | GBR | NAO |  | SCF | 18 | 1,109,254 | 65,472 | 174,840 |
| GBR NAO PGP0010 NGI | GBR | NAO |  | SCF | 88 | 1,454,045 | 16,838 | 708,662 |
| LTU NAO DFN1012 NGI | LTU | NAO |  | SCF | 10 | 348,315 | 37,227 | 147,130 |
| LTU NAO PG 0010 NGI | LTU | NAO |  | SCF | 63 | 162,216 | 2,486 | 168,374 |
| LVA NAO PGP0010 NGI | LVA | NAO |  | SCF | 380 | 4,273,046 | 6,226 | 950,733 |
| NLD NAO PG 0010 NGI | NLD | NAO |  | SCF | 166 | 11,455,435 | 68,955 | 1,920,423 |
| NLD NAO PG 1012 NGI | NLD | NAO |  | SCF | 16 | 1,094,216 | 69,761 | 184,154 |
| PRT NAO DFN0010 NGI | PRT | NAO |  | SCF | 482 | 6,563,721 | 13,894 | 3,588,930 |
| PRT NAO DFN1012 NGI | PRT | NAO |  | SCF | 26 | 2,213,970 | 85,687 | 1,388,023 |
| PRT NAO FPO0010 NGI | PRT | NAO |  | SCF | 157 | 4,368,965 | 27,912 | 5,030,297 |
| PRT NAO FPO1012 NGI | PRT | NAO |  | SCF | 53 | 4,447,564 | 84,291 | 3,636,328 |
| PRT NAO HOK0010 NGI | PRT | NAO |  | SCF | 182 | 1,575,576 | 8,658 | 1,828,990 |
| PRT NAO HOK1012 NGI | PRT | NAO |  | SCF | 13 | 1,034,444 | 79,991 | 933,785 |
| PRT NAO PGP0010 NGI | PRT | NAO |  | SCF | 1,759 | 30,439,550 | 17,329 | 22,557,501 |
| PRT NAO PGP1012 NGI | PRT | NAO |  | SCF | 14 | 1,073,066 | 78,969 | 395,731 |
| PRT NAO PMP0010 NGI | PRT | NAO |  | SCF | 60 | 1,176,770 | 19,823 | 689,083 |
| SWE NAO DFN0010 NGI | SWE | NAO |  | SCF | 642 | 9,839,650 | 15,333 | 2,805,328 |
| SWE NAO DFN1012 NGI | SWE | NAO |  | SCF | 145 | 7,477,433 | 51,424 | 1,595,383 |
| PRT NAO HOK0010 P2 | PRT | NAO | Madeira | SCF | 58 | 935,756 | 16,141 | 652,614 |
| PRT NAO DFN0010 P3 | PRT | NAO | Azores | SCF | 43 | 629,267 | 14,442 | 540,881 |
| PRT NAO HOK0010 P3 | PRT | NAO | Azores | SCF | 392 | 10,457,232 | 26,391 | 6,022,341 |
| PRT NAO HOK1012 P3 | PRT | NAO | Azores | SCF | 70 | 6,019,984 | 85,458 | 3,721,379 |
| PRT NAO PGP0010 P3 | PRT | NAO | Azores | SCF | 14 | 421,852 | 33,303 | 201,721 |
| BGR MBS DFN1218 NGI | BGR | MBS |  | LSF | 10 | 596,602 | 60,996 | - 6,883 |
| BGR MBS PMP1218 NGI | BGR | MBS |  | LSF | 27 | 2,936,623 | 106,796 | -17,118 |
| BGR MBS PMP1824 NGI | BGR | MBS |  | LSF | 7 | 917,529 | 128,181 | 98,148 |
| BGR MBS PS 0006 NGI | BGR | MBS |  | LSF | 23 | 30,438 | 1,314 | 4,915 |
| BGR MBS TM 1218 NGI | BGR | MBS |  | LSF | 21 | 2,223,214 | 113,030 | 38,224 |
| BGR MBS TM 1824 NGI | BGR | MBS |  | LSF | 6 | 830,336 | 134,274 | 188,503 |
| BGR MBS TM 2440 NGI | BGR | MBS |  | LSF | 11 | 2,953,860 | 260,990 | 743,391 |
| CYP MBS DTS2440 NGI | CYP | MBS |  | LSF | 7 | 7,608,076 | 1,186,789 | -1,186,299 |
| CYP MBS PGP1218 NGI | CYP | MBS |  | LSF | 22 | 7,790,421 | 345,692 | -1,108,813 |
| ESP MBS DTS0612 NGI | ESP | MBS |  | LSF | 24 | 308,387 | 13,377 | 1,098,593 |
| ESP MBS DTS1218 NGI | ESP | MBS |  | LSF | 171 | 8,549,501 | 49,917 | 10,596,469 |
| ESP MBS DTS1824 NGI | ESP | MBS |  | LSF | 370 | 35,077,441 | 92,072 | 25,986,019 |
| ESP MBS DTS2440 NGI | ESP | MBS |  | LSF | 158 | 33,847,305 | 205,731 | 16,295,499 |
| ESP MBS HOK1218 NGI | ESP | MBS |  | LSF | 86 | 4,527,751 | 51,481 | 4,025,713 |
| ESP MBS HOK1824 NGI | ESP | MBS |  | LSF | 24 | 3,219,562 | 129,710 | 2,926,403 |
| ESP MBS PS 0612 NGI | ESP | MBS |  | LSF | 24 | 261,748 | 10,774 | 1,270,687 |
| ESP MBS PS 1218 NGI | ESP | MBS |  | LSF | 100 | 4,327,525 | 42,928 | 14,248,304 |
| ESP MBS PS 1824 NGI | ESP | MBS |  | LSF | 95 | 10,606,972 | 111,202 | 18,413,994 |
| ESP MBS PS 2440 NGI | ESP | MBS |  | LSF | 24 | 9,944,601 | 436,836 | 10,581,668 |
| ITA MBS DRB1218 NGI | ITA | MBS |  | LSF | 697 | 44,947,530 | 64,376 | 28,672,228 |
| ITA MBS DTS0612 NGI | ITA | MBS |  | LSF | 169 | 4,788,216 | 28,562 | 4,636,239 |
| ITA MBS DTS1218 NGI | ITA | MBS |  | LSF | 1,315 | 87,154,693 | 65,943 | 83,334,650 |
| ITA MBS DTS1824 NGI | ITA | MBS |  | LSF | 674 | 156,076,898 | 230,754 | 48,585,388 |
| ITA MBS DTS2440 NGI | ITA | MBS |  | LSF | 216 | 124,645,171 | 570,933 | 15,522,118 |
| ITA MBS HOK1218 NGI | ITA | MBS |  | LSF | 133 | 10,249,728 | 76,600 | 9,690,983 |
| ITA MBS HOK1824 NGI | ITA | MBS |  | LSF | 47 | 13,192,923 | 280,118 | 3,818,576 |
| ITA MBS PGP1218 NGI | ITA | MBS |  | LSF | 386 | 31,603,109 | 81,685 | 19,623,598 |
| ITA MBS PMP1218 NGI | ITA | MBS |  | LSF | 37 | 1,540,678 | 41,087 | 1,561,478 |
| ITA MBS PS 1824 NGI | ITA | MBS |  | LSF | 47 | 9,220,427 | 198,954 | 7,450,333 |
| ITA MBS PS 2440 NGI | ITA | MBS |  | LSF | 50 | 27,807,932 | 558,729 | 5,743,800 |
| ITA MBS TBB1218 NGI | ITA | MBS |  | LSF | 11 | 779,379 | 71,001 | 877,901 |
| ITA MBS TBB1824 NGI | ITA | MBS |  | LSF | 28 | 6,335,906 | 220,379 | 1,734,172 |
| ITA MBS TBB2440 NGI | ITA | MBS |  | LSF | 25 | 9,708,372 | 397,660 | 2,426,242 |
| ITA MBS TM 1218 NGI | ITA | MBS |  | LSF | 34 | 1,819,635 | 54,037 | 5,241,578 |
| ITA MBS TM 1824 NGI | ITA | MBS |  | LSF | 43 | 8,570,261 | 199,242 | 4,150,048 |
| ITA MBS TM 2440 NGI | ITA | MBS |  | LSF | 69 | 23,879,509 | 342,359 | 7,591,544 |
| MLT MBS DTS1824 NGI | MLT | MBS |  | LSF | 11 | 13,023,317 | 1,113,250 | -215,358 |
| MLT MBS DTS2440 NGI | MLT | MBS |  | LSF | 4 | 9,744,613 | 2,296,484 | -842,809 |
| MLT MBS HOK1218 NGI | MLT | MBS |  | LSF | 15 | 3,358,828 | 230,926 | 426,875 |
| MLT MBS HOK1824 NGI | MLT | MBS |  | LSF | 15 | 8,886,375 | 585,993 | 114,213 |
| MLT MBS MGO0612 NGI | MLT | MBS |  | LSF | 14 | 1,061,288 | 82,337 | 8,981 |
| MLT MBS MGO1218 NGI | MLT | MBS |  | LSF | 8 | 1,810,187 | 223,799 | 211,662 |
| PRT MBS FPO2440 NGI | PRT | MBS |  | LSF | 2 | 1,454,750 | 727,375 | 393,867 |
| SVN MBS DTS1218 NGI | SVN | MBS |  | LSF | 14 | 1,221,862 | 85,634 | 538,389 |
| SVN MBS PS 1218 NGI | SVN | MBS |  | LSF | 4 | 318,099 | 89,533 | 376,608 |
| IRL NAO DRB2440 | IRL | NAO |  | LSF | 7 | 5,486,312 | 821,452 | 5,762,893 |
| IRL NAO DTS1218 | IRL | NAO |  | LSF | 48 | 10,974,701 | 229,185 | 3,609,745 |
| IRL NAO DTS1824 | IRL | NAO |  | LSF | 66 | 45,155,496 | 684,839 | 13,044,267 |
| IRL NAO DTS2440 | IRL | NAO |  | LSF | 36 | 49,444,488 | 1,369,852 | 11,392,769 |
| IRL NAO FPO1218 | IRL | NAO |  | LSF | 22 | 3,347,556 | 145,645 | 2,674,725 |
| IRL NAO TM 2440 | IRL | NAO |  | LSF | 12 | 47,291,458 | 4,024,539 | 3,437,002 |
| IRL NAO TM 40XX | IRL | NAO |  | LSF | 20 | 300,342,705 | 14,789,153 | 6,455,590 |
| POL NAO DFN1218 | POL | NAO |  | LSF | 33 | 6,974,332 | 211,283 | 774,178 |
| POL NAO TM 2440 | POL | NAO |  | LSF | 47 | 32,550,317 | 690,135 | 6,434,640 |
| PRT NAO DTS40XX IWE | PRT | NAO |  | LSF | 12 | 49,783,960 | 4,147,584 | 22,232,458 |
| BEL NAO DTS2440 NGI | BEL | NAO |  | LSF | 11 | 8,431,912 | 762,417 | 3,024,793 |
| BEL NAO PMP1824 NGI | BEL | NAO |  | LSF | 4 | 2,503,311 | 568,063 | 622,277 |
| BEL NAO TBB1824 NGI | BEL | NAO |  | LSF | 33 | 14,996,546 | 441,245 | 3,683,559 |
| BEL NAO TBB2440 NGI | BEL | NAO |  | LSF | 33 | 34,292,717 | 1,030,966 | 18,559,297 |
| DEU NAO DFN1218 NGI | DEU | NAO |  | LSF | 10 | 716,392 | 73,295 | 1,108,504 |
| DEU NAO DFN2440 NGI | DEU | NAO |  | LSF | 8 | 3,041,270 | 403,327 | 908,635 |
| DEU NAO DTS1012 NGI | DEU | NAO |  | LSF | 13 | 629,897 | 47,404 | 183,758 |
| DEU NAO DTS1218 NGI | DEU | NAO |  | LSF | 32 | 2,891,352 | 89,993 | 1,026,267 |
| DEU NAO DTS1824 NGI | DEU | NAO |  | LSF | 22 | 7,435,804 | 334,910 | 4,922,920 |
| DEU NAO DTS2440 NGI | DEU | NAO |  | LSF | 12 | 10,162,015 | 849,506 | 5,558,373 |
| DEU NAO DTS40XX NGI | DEU | NAO |  | LSF | 7 | 39,047,369 | 5,368,321 | 11,571,287 |
| DEU NAO TBB1012 NGI | DEU | NAO |  | LSF | 15 | 295,128 | 19,726 | 237,395 |
| DEU NAO TBB1218 NGI | DEU | NAO |  | LSF | 125 | 10,568,459 | 84,431 | 13,565,253 |
| DEU NAO TBB1824 NGI | DEU | NAO |  | LSF | 63 | 11,128,849 | 176,647 | 8,581,965 |
| DEU NAO TBB2440 NGI | DEU | NAO |  | LSF | 9 | 5,356,947 | 626,402 | 2,683,898 |
| DNK NAO DRB1012 NGI | DNK | NAO |  | LSF | 22 | 8,048,752 | 377,345 | 1,940,472 |
| DNK NAO DRB1218 NGI | DNK | NAO |  | LSF | 28 | 10,983,741 | 408,964 | 2,297,536 |
| DNK NAO DTS0010 NGI | DNK | NAO |  | LSF | 11 | 1,994,215 | 188,173 | 183,726 |
| DNK NAO DTS1218 NGI | DNK | NAO |  | LSF | 144 | 49,389,854 | 343,057 | 14,878,143 |
| DNK NAO DTS1824 NGI | DNK | NAO |  | LSF | 64 | 52,304,290 | 834,002 | 18,118,887 |
| DNK NAO DTS2440 NGI | DNK | NAO |  | LSF | 39 | 62,902,373 | 1,652,739 | 21,712,658 |
| DNK NAO DTS40XX NGI | DNK | NAO |  | LSF | 21 | 96,988,945 | 4,190,592 | 37,700,747 |
| DNK NAO PGP1218 NGI | DNK | NAO |  | LSF | 41 | 15,730,459 | 385,604 | 4,414,768 |
| DNK NAO PMP1218 NGI | DNK | NAO |  | LSF | 42 | 12,161,683 | 294,945 | 3,178,754 |
| DNK NAO PMP1824 NGI | DNK | NAO |  | LSF | 15 | 16,024,017 | 1,068,588 | 6,557,041 |
| DNK NAO TBB1218 NGI | DNK | NAO |  | LSF | 12 | 6,051,958 | 518,019 | 1,559,039 |
| DNK NAO TBB1824 NGI | DNK | NAO |  | LSF | 16 | 13,571,041 | 848,444 | 3,037,578 |
| ESP NAO DFN1218 NGI | ESP | NAO |  | LSF | 129 | 4,687,146 | 38,745 | 7,257,543 |
| ESP NAO DFN1824 NGI | ESP | NAO |  | LSF | 30 | 2,541,677 | 85,205 | 6,739,057 |
| ESP NAO DTS1218 NGI | ESP | NAO |  | LSF | 69 | 3,509,362 | 49,799 | 6,295,048 |
| ESP NAO DTS1824 NGI | ESP | NAO |  | LSF | 87 | 8,886,029 | 97,714 | 9,567,780 |
| ESP NAO DTS2440 NGI | ESP | NAO |  | LSF | 176 | 68,442,120 | 370,059 | 55,243,706 |
| ESP NAO DTS40XX NGI | ESP | NAO |  | LSF | 26 | 23,388,498 | 846,763 | 39,773,922 |
| ESP NAO HOK1218 NGI | ESP | NAO |  | LSF | 87 | 4,423,109 | 50,016 | 6,461,533 |
| ESP NAO HOK1824 NGI | ESP | NAO |  | LSF | 45 | 4,579,444 | 100,633 | 9,366,652 |
| ESP NAO PS 1012 NGI | ESP | NAO |  | LSF | 24 | 283,826 | 12,317 | 1,391,659 |
| ESP NAO PS 1218 NGI | ESP | NAO |  | LSF | 129 | 5,317,218 | 40,997 | 16,558,152 |
| ESP NAO PS 1824 NGI | ESP | NAO |  | LSF | 102 | 10,500,073 | 102,400 | 20,213,498 |
| ESP NAO PS 2440 NGI | ESP | NAO |  | LSF | 99 | 20,735,134 | 203,507 | 38,635,182 |
| EST NAO TM 1218 NGI | EST | NAO |  | LSF | 11 | 384,013 | 29,128 | 87,562 |
| EST NAO TM 2440 NGI | EST | NAO |  | LSF | 32 | 10,761,845 | 350,453 | 5,001,257 |
| FIN NAO TM 1218 NGI | FIN | NAO |  | LSF | 24 | 2,086,051 | 88,309 | 196,894 |
| FIN NAO TM 1824 NGI | FIN | NAO |  | LSF | 14 | 3,168,248 | 231,549 | 986,216 |
| FIN NAO TM 2440 NGI | FIN | NAO |  | LSF | 20 | 14,816,357 | 753,120 | 726,414 |
| GBR NAO DFN2440 NGI | GBR | NAO |  | LSF | 15 | 4,307,676 | 292,811 | 7,125,809 |
| GBR NAO DRB0010 NGI | GBR | NAO |  | LSF | 110 | 4,365,397 | 40,059 | 2,346,668 |
| GBR NAO DRB1012 NGI | GBR | NAO |  | LSF | 29 | 2,555,423 | 86,745 | 1,915,124 |
| GBR NAO DRB1218 NGI | GBR | NAO |  | LSF | 88 | 14,557,742 | 167,046 | 9,391,084 |
| GBR NAO DRB1824 NGI | GBR | NAO |  | LSF | 22 | 8,363,401 | 370,799 | 6,755,628 |
| GBR NAO DRB2440 NGI | GBR | NAO |  | LSF | 29 | 9,970,307 | 357,222 | 11,224,299 |
| GBR NAO DTS0010 NGI | GBR | NAO |  | LSF | 280 | 12,652,682 | 45,272 | 6,740,882 |
| GBR NAO DTS1012 NGI | GBR | NAO |  | LSF | 96 | 6,454,429 | 68,116 | 4,538,494 |
| GBR NAO DTS1218 NGI | GBR | NAO |  | LSF | 232 | 23,984,411 | 103,766 | 22,278,595 |
| GBR NAO DTS1824 NGI | GBR | NAO |  | LSF | 190 | 81,769,423 | 427,423 | 41,845,811 |
| GBR NAO DTS2440 NGI | GBR | NAO |  | LSF | 96 | 63,968,164 | 658,098 | 57,197,275 |
| GBR NAO DTS40XX NGI | GBR | NAO |  | LSF | 11 | 23,834,061 | 2,186,981 | 17,162,098 |
| GBR NAO FPO1218 NGI | GBR | NAO |  | LSF | 75 | 10,016,925 | 134,599 | 10,311,242 |
| GBR NAO FPO1824 NGI | GBR | NAO |  | LSF | 13 | 7,987,543 | 594,574 | 5,596,232 |
| GBR NAO HOK2440 NGI | GBR | NAO |  | LSF | 15 | 3,356,077 | 245,281 | 8,347,557 |
| GBR NAO MGP0010 NGI | GBR | NAO |  | LSF | 16 | 724,457 | 44,948 | 380,207 |
| GBR NAO MGP1218 NGI | GBR | NAO |  | LSF | 23 | 2,018,875 | 107,239 | 1,499,517 |
| GBR NAO TBB0010 NGI | GBR | NAO |  | LSF | 28 | 750,091 | 31,932 | 256,920 |
| GBR NAO TBB1218 NGI | GBR | NAO |  | LSF | 23 | 1,887,663 | 87,406 | 460,627 |
| GBR NAO TBB1824 NGI | GBR | NAO |  | LSF | 17 | 7,671,290 | 439,430 | 5,159,535 |
| GBR NAO TBB2440 NGI | GBR | NAO |  | LSF | 34 | 14,122,589 | 403,066 | 9,258,613 |
| GBR NAO TM 40XX NGI | GBR | NAO |  | LSF | 31 | 170,911,648 | 5,615,447 | 132,012,803 |
| LTU NAO DTS2440 NGI | LTU | NAO |  | LSF | 18 | 3,314,836 | 181,368 | 618,346 |
| LTU NAO TM 2440 NGI | LTU | NAO |  | LSF | 5 | 2,852,494 | 624,352 | 890,442 |
| LVA NAO TM 1218 NGI | LVA | NAO |  | LSF | 16 | 2,942,215 | 167,781 | 297,932 |
| LVA NAO TM 2440 NGI | LVA | NAO |  | LSF | 51 | 15,708,392 | 290,144 | 6,451,422 |
| NLD NAO DFN1218 NGI | NLD | NAO |  | LSF | 14 | 1,184,470 | 78,830 | 260,258 |
| NLD NAO DFN1824 NGI | NLD | NAO |  | LSF | 12 | 2,294,898 | 201,719 | 464,171 |
| NLD NAO DRB2440 NGI | NLD | NAO |  | LSF | 9 | 2,598,967 | 317,073 | 3,350,796 |
| NLD NAO DTS0010 NGI | NLD | NAO |  | LSF | 13 | 1,453,423 | 109,624 | -106,752 |
| NLD NAO DTS1824 NGI | NLD | NAO |  | LSF | 12 | 3,707,779 | 332,202 | 2,680,304 |
| NLD NAO DTS2440 NGI | NLD | NAO |  | LSF | 22 | 13,266,033 | 603,267 | 9,054,308 |
| NLD NAO TBB0010 NGI | NLD | NAO |  | LSF | 18 | 2,065,691 | 111,928 | 98,582 |
| NLD NAO TBB1218 NGI | NLD | NAO |  | LSF | 17 | 3,189,215 | 182,744 | 561,008 |
| NLD NAO TBB1824 NGI | NLD | NAO |  | LSF | 156 | 40,974,655 | 262,093 | 31,791,095 |
| NLD NAO TBB2440 NGI | NLD | NAO |  | LSF | 29 | 17,333,646 | 582,378 | 9,565,636 |
| NLD NAO TBB40XX NGI | NLD | NAO |  | LSF | 58 | 51,299,251 | 857,131 | 41,026,810 |
| NLD NAO TM 40XX NGI | NLD | NAO |  | LSF | 11 | 192,744,568 | 17,335,625 | 18,537,942 |
| PRT NAO DFN1218 NGI | PRT | NAO |  | LSF | 72 | 12,692,928 | 173,517 | 5,530,414 |
| PRT NAO DFN1824 NGI | PRT | NAO |  | LSF | 26 | 9,364,053 | 356,645 | 2,276,586 |
| PRT NAO DRB0010 NGI | PRT | NAO |  | LSF | 50 | 1,414,132 | 28,355 | 341,613 |
| PRT NAO DRB1012 NGI | PRT | NAO |  | LSF | 23 | 1,987,815 | 86,905 | 192,457 |
| PRT NAO DRB1218 NGI | PRT | NAO |  | LSF | 15 | 2,006,671 | 135,582 | 869,194 |
| PRT NAO DTS0010 NGI | PRT | NAO |  | LSF | 5 | 485,172 | 104,160 | 677,319 |
| PRT NAO DTS1218 NGI | PRT | NAO |  | LSF | 9 | 1,936,377 | 215,319 | 1,195,701 |
| PRT NAO DTS1824 NGI | PRT | NAO |  | LSF | 8 | 4,005,005 | 484,767 | 1,683,514 |
| PRT NAO DTS2440 NGI | PRT | NAO |  | LSF | 62 | 47,315,977 | 762,899 | 11,834,125 |
| PRT NAO FPO1218 NGI | PRT | NAO |  | LSF | 54 | 8,785,231 | 162,168 | 5,821,154 |
| PRT NAO FPO1824 NGI | PRT | NAO |  | LSF | 7 | 2,869,909 | 385,579 | 1,007,662 |
| PRT NAO HOK1218 NGI | PRT | NAO |  | LSF | 23 | 4,069,059 | 178,428 | 4,180,039 |
| PRT NAO HOK1824 NGI | PRT | NAO |  | LSF | 25 | 10,846,598 | 429,385 | 7,384,039 |
| PRT NAO HOK2440 NGI | PRT | NAO |  | LSF | 18 | 12,898,000 | 706,161 | 4,145,691 |
| PRT NAO MGO0010 NGI | PRT | NAO |  | LSF | 33 | 905,528 | 27,868 | 996,974 |
| PRT NAO MGO1012 NGI | PRT | NAO |  | LSF | 7 | 398,510 | 59,957 | 338,458 |
| PRT NAO PGP1218 NGI | PRT | NAO |  | LSF | 37 | 6,672,621 | 182,358 | 3,182,133 |
| PRT NAO PS 0010 NGI | PRT | NAO |  | LSF | 25 | 1,066,085 | 43,560 | 1,077,682 |
| PRT NAO PS 1012 NGI | PRT | NAO |  | LSF | 27 | 2,352,993 | 86,398 | 2,850,936 |
| PRT NAO PS 1218 NGI | PRT | NAO |  | LSF | 37 | 4,790,059 | 130,400 | 5,468,310 |
| PRT NAO PS 1824 NGI | PRT | NAO |  | LSF | 51 | 18,635,932 | 364,383 | 18,175,589 |
| PRT NAO PS 2440 NGI | PRT | NAO |  | LSF | 18 | 9,982,214 | 548,910 | 8,746,301 |
| PRT NAO TBB0010 NGI | PRT | NAO |  | LSF | 55 | 1,144,218 | 20,813 | 498,178 |
| PRT NAO TBB1012 NGI | PRT | NAO |  | LSF | 8 | 683,198 | 88,176 | 253,458 |
| SWE NAO DFN1218 NGI | SWE | NAO |  | LSF | 20 | 2,327,137 | 115,568 | 382,576 |
| SWE NAO DTS1012 NGI | SWE | NAO |  | LSF | 77 | 5,515,529 | 71,093 | 2,535,140 |
| SWE NAO DTS1218 NGI | SWE | NAO |  | LSF | 83 | 10,386,921 | 122,847 | 6,521,246 |
| SWE NAO DTS1824 NGI | SWE | NAO |  | LSF | 49 | 17,943,876 | 368,485 | 5,132,125 |
| SWE NAO DTS2440 NGI | SWE | NAO |  | LSF | 49 | 63,944,205 | 1,295,797 | 25,101,712 |
| PRT NAO HOK1218 P2 | PRT | NAO | Madeira | LSF | 21 | 2,702,213 | 125,498 | 4,303,126 |
| PRT NAO HOK2440 P2 | PRT | NAO | Madeira | LSF | 6 | 5,824,917 | 1,010,225 | 468,897 |
| PRT NAO MGP1824 P2 | PRT | NAO | Madeira | LSF | 4 | 669,983 | 186,604 | 256,639 |
| PRT NAO HOK1218 P3 | PRT | NAO | Azores | LSF | 40 | 4,354,610 | 107,986 | 3,537,111 |
| PRT NAO HOK2440 P3 | PRT | NAO | Azores | LSF | 24 | 16,997,034 | 698,831 | 4,342,246 |
| PRT NAO PS 0010 P3 | PRT | NAO | Azores | LSF | 28 | 584,160 | 19,906 | 327,272 |
| PRT NAO PS 1012 P3 | PRT | NAO | Azores | LSF | 12 | 965,039 | 79,515 | 865,232 |
| PRT OFR HOK2440 IWE | PRT | OFR |  | DWF | 12 | 13,118,281 | 1,076,945 | 2,513,660 |
| PRT OFR HOK40XX IWE | PRT | OFR |  | DWF | 5 | 9,039,002 | 1,691,708 | 1,574,365 |
| LTU OFR TM 40XX NEU | LTU | OFR |  | DWF | 10 | 61,074,395 | 6,044,804 | 5,067,087 |
| ESP OFR DTS2440 NGI | ESP | OFR |  | DWF | 52 | 16,536,049 | 306,315 | 29,991,328 |
| ESP OFR DTS40XX NGI | ESP | OFR |  | DWF | 31 | 24,862,203 | 802,496 | 46,318,485 |
| ESP OFR HOK2440 NGI | ESP | OFR |  | DWF | 98 | 28,595,427 | 291,141 | 32,029,727 |
| ESP OFR HOK40XX NGI | ESP | OFR |  | DWF | 29 | 17,566,329 | 584,211 | 11,587,616 |
| ESP OFR PS 40XX NGI | ESP | OFR |  | DWF | 33 | 109,455,051 | 3,521,250 | 106,947,690 |
| TOTAL | | | | | 34,039 | 3,871,856,770 | 113,749 | 1,986,744,784 |
