# Supplementary material for: Characterising investments in EU fisheries and defining their desirability
Source: Fish Res. 2020 Jan;221:105396. doi: 10.1016/j.fishres.2019.105396 (PMC6853412; doi:10.1016/j.fishres.2019.105396)

**Supplementary material 2**

Table SM2.2a: Evolution of annual between-year investment and their outputs (changes in NVA) for the EU fleet by sea basin (A27: Northeast Atlantic; A37: Mediterranean and Black Sea) and scale (SCF: small-scale fleet; LSF: large=scale fleet). Values represent the number of fleets (a) and the number of vessels in each fleet (b) exhibiting the different types of investment behaviour between the year indicated and the preceding year.

SCF A37

| Capital | Number of vessels | Average value per vessel | NVA | 2009 | 2010 | 2011 | 2012 | 2013 | 2014 | 2015 | 2016 |
| --- | --- | --- | --- | --- | --- | --- | --- | --- | --- | --- | --- |
| Increase | Increase | Increase | Increase | 2 | 1 | 2 | 3 | 0 | 0 | 0 | 0 |
| Increase | Increase | Increase | Decrease | 3 | 4 | 0 | 1 | 4 | 3 | 5 | 3 |
| Increase | Stable | Increase | Increase | 0 | 0 | 1 | 0 | 0 | 0 | 1 | 0 |
| Increase | Stable | Increase | Decrease | 0 | 0 | 1 | 0 | 0 | 0 | 2 | 1 |
| Increase | Increase | Decrease | Increase | 5 | 5 | 2 | 3 | 1 | 0 | 3 | 1 |
| Increase | Increase | Decrease | Decrease | 1 | 4 | 4 | 2 | 0 | 0 | 3 | 0 |
| Increase | Decrease | Increase | Increase | 2 | 1 | 0 | 2 | 0 | 0 | 2 | 2 |
| Increase | Decrease | Increase | Decrease | 1 | 1 | 2 | 0 | 0 | 4 | 1 | 1 |
| Decrease | Decrease | Increase | Increase | 0 | 0 | 0 | 0 | 2 | 1 | 0 | 0 |
| Decrease | Decrease | Increase | Decrease | 2 | 1 | 1 | 1 | 1 | 3 | 1 | 3 |
| Decrease | Increase | Decrease | Increase | 0 | 0 | 0 | 0 | 4 | 0 | 0 | 1 |
| Decrease | Increase | Decrease | Decrease | 1 | 0 | 0 | 0 | 4 | 4 | 0 | 1 |
| Decrease | Stable | Decrease | Increase | 1 | 0 | 0 | 0 | 2 | 2 | 1 | 0 |
| Decrease | Stable | Decrease | Decrease | 0 | 0 | 0 | 0 | 0 | 0 | 0 | 0 |
| Decrease | Decrease | Decrease | Increase | 2 | 2 | 4 | 5 | 1 | 5 | 2 | 3 |
| Decrease | Decrease | Decrease | Decrease | 2 | 3 | 5 | 5 | 3 | 0 | 1 | 6 |

LSF A37

| Capital | Number of vessels | Average value per vessel | NVA | 2009 | 2010 | 2011 | 2012 | 2013 | 2014 | 2015 | 2016 |
| --- | --- | --- | --- | --- | --- | --- | --- | --- | --- | --- | --- |
| Increase | Increase | Increase | Increase | 2 | 2 | 1 | 1 | 1 | 3 | 2 | 0 |
| Increase | Increase | Increase | Decrease | 3 | 3 | 2 | 2 | 4 | 2 | 1 | 5 |
| Increase | Stable | Increase | Increase | 0 | 0 | 1 | 0 | 0 | 1 | 0 | 1 |
| Increase | Stable | Increase | Decrease | 0 | 0 | 1 | 0 | 1 | 1 | 1 | 1 |
| Increase | Increase | Decrease | Increase | 1 | 0 | 0 | 2 | 1 | 4 | 0 | 1 |
| Increase | Increase | Decrease | Decrease | 2 | 1 | 1 | 6 | 0 | 3 | 2 | 2 |
| Increase | Decrease | Increase | Increase | 4 | 5 | 1 | 4 | 3 | 2 | 3 | 2 |
| Increase | Decrease | Increase | Decrease | 2 | 5 | 1 | 2 | 1 | 2 | 3 | 5 |
| Decrease | Decrease | Increase | Increase | 5 | 3 | 5 | 5 | 6 | 2 | 3 | 0 |
| Decrease | Decrease | Increase | Decrease | 1 | 6 | 1 | 0 | 1 | 1 | 4 | 3 |
| Decrease | Increase | Decrease | Increase | 6 | 3 | 1 | 0 | 5 | 3 | 3 | 2 |
| Decrease | Increase | Decrease | Decrease | 0 | 1 | 4 | 3 | 1 | 3 | 2 | 7 |
| Decrease | Stable | Decrease | Increase | 5 | 2 | 0 | 3 | 1 | 0 | 0 | 1 |
| Decrease | Stable | Decrease | Decrease | 0 | 2 | 2 | 2 | 0 | 0 | 2 | 0 |
| Decrease | Decrease | Decrease | Increase | 6 | 5 | 10 | 2 | 8 | 6 | 6 | 4 |
| Decrease | Decrease | Decrease | Decrease | 2 | 1 | 8 | 7 | 6 | 6 | 7 | 5 |

SCF A27

| Capital | Number of vessels | Average value per vessel | NVA | 2009 | 2010 | 2011 | 2012 | 2013 | 2014 | 2015 | 2016 |
| --- | --- | --- | --- | --- | --- | --- | --- | --- | --- | --- | --- |
| Increase | Increase | Increase | Increase | 3 | 7 | 1 | 1 | 1 | 2 | 2 | 1 |
| Increase | Increase | Increase | Decrease | 5 | 1 | 1 | 4 | 1 | 4 | 3 | 2 |
| Increase | Stable | Increase | Increase | 0 | 2 | 0 | 2 | 0 | 3 | 3 | 0 |
| Increase | Stable | Increase | Decrease | 0 | 1 | 2 | 1 | 3 | 2 | 2 | 2 |
| Increase | Increase | Decrease | Increase | 6 | 4 | 1 | 3 | 0 | 1 | 1 | 0 |
| Increase | Increase | Decrease | Decrease | 4 | 2 | 1 | 2 | 0 | 1 | 4 | 4 |
| Increase | Decrease | Increase | Increase | 3 | 1 | 3 | 0 | 1 | 4 | 2 | 2 |
| Increase | Decrease | Increase | Decrease | 4 | 2 | 1 | 2 | 2 | 1 | 3 | 4 |
| Decrease | Decrease | Increase | Increase | 1 | 2 | 2 | 3 | 4 | 4 | 3 | 3 |
| Decrease | Decrease | Increase | Decrease | 2 | 1 | 4 | 4 | 1 | 3 | 2 | 1 |
| Decrease | Increase | Decrease | Increase | 1 | 1 | 6 | 1 | 1 | 1 | 1 | 5 |
| Decrease | Increase | Decrease | Decrease | 3 | 2 | 2 | 4 | 1 | 4 | 1 | 1 |
| Decrease | Stable | Decrease | Increase | 1 | 3 | 2 | 2 | 4 | 4 | 3 | 2 |
| Decrease | Stable | Decrease | Decrease | 3 | 1 | 1 | 1 | 5 | 0 | 3 | 2 |
| Decrease | Decrease | Decrease | Increase | 3 | 12 | 10 | 11 | 13 | 5 | 3 | 6 |
| Decrease | Decrease | Decrease | Decrease | 6 | 3 | 8 | 4 | 8 | 6 | 9 | 10 |

LSF A27

| Capital | Number of vessels | Average value per vessel | NVA | 2009 | 2010 | 2011 | 2012 | 2013 | 2014 | 2015 | 2016 |
| --- | --- | --- | --- | --- | --- | --- | --- | --- | --- | --- | --- |
| Increase | Increase | Increase | Increase | 19 | 5 | 5 | 2 | 8 | 11 | 5 | 3 |
| Increase | Increase | Increase | Decrease | 8 | 14 | 6 | 8 | 11 | 18 | 13 | 18 |
| Increase | Stable | Increase | Increase | 7 | 2 | 5 | 5 | 8 | 3 | 4 | 3 |
| Increase | Stable | Increase | Decrease | 5 | 4 | 3 | 7 | 3 | 6 | 7 | 4 |
| Increase | Increase | Decrease | Increase | 1 | 5 | 4 | 5 | 8 | 3 | 2 | 0 |
| Increase | Increase | Decrease | Decrease | 5 | 5 | 5 | 5 | 2 | 6 | 4 | 5 |
| Increase | Decrease | Increase | Increase | 17 | 6 | 10 | 10 | 9 | 8 | 9 | 7 |
| Increase | Decrease | Increase | Decrease | 3 | 11 | 2 | 12 | 4 | 11 | 12 | 10 |
| Decrease | Decrease | Increase | Increase | 6 | 9 | 6 | 7 | 5 | 2 | 6 | 8 |
| Decrease | Decrease | Increase | Decrease | 4 | 12 | 5 | 5 | 6 | 4 | 8 | 6 |
| Decrease | Increase | Decrease | Increase | 2 | 5 | 5 | 3 | 10 | 2 | 3 | 4 |
| Decrease | Increase | Decrease | Decrease | 6 | 5 | 11 | 5 | 7 | 6 | 11 | 9 |
| Decrease | Stable | Decrease | Increase | 7 | 5 | 5 | 8 | 11 | 6 | 6 | 7 |
| Decrease | Stable | Decrease | Decrease | 5 | 7 | 9 | 8 | 7 | 14 | 9 | 6 |
| Decrease | Decrease | Decrease | Increase | 18 | 15 | 22 | 19 | 21 | 11 | 8 | 14 |
| Decrease | Decrease | Decrease | Decrease | 15 | 18 | 25 | 19 | 8 | 17 | 21 | 24 |

Table SM2.2b: Evolution of annual between-year investment and their outputs (changes in NVA) for the EU fishing vessels by fleet, by sea basin (A27: Northeast Atlantic; A37: Mediterranean and Black Sea) and scale (SCF: small-scale fleet; LSF: large=scale fleet). Values represent the number of vessels in each fleet exhibiting the different types of investment behaviour between the year indicated and the preceding year.

SCF A37

| Capital | Number of vessels | Average value per vessel | NVA | 2009 | 2010 | 2011 | 2012 | 2013 | 2014 | 2015 | 2016 |
| --- | --- | --- | --- | --- | --- | --- | --- | --- | --- | --- | --- |
| Increase | Increase | Increase | Increase | 317 | 5,361 | 5,426 | 600 | - | - | - | - |
| Increase | Increase | Increase | Decrease | 2,703 | 579 | - | 33 | 278 | 74 | 1,014 | 127 |
| Increase | Stable | Increase | Increase | - | - | 44 | - | - | - | 35 | - |
| Increase | Stable | Increase | Decrease | - | - | 456 | - | - | - | 111 | 41 |
| Increase | Increase | Decrease | Increase | 493 | 460 | 53 | 428 | 43 | - | 804 | 154 |
| Increase | Increase | Decrease | Decrease | 368 | 766 | 193 | 564 | - | - | 222 | - |
| Increase | Decrease | Increase | Increase | 479 | 2,461 | - | 179 | - | - | 73 | 122 |
| Increase | Decrease | Increase | Decrease | 5,338 | 35 | 183 | - | - | 823 | 2,214 | 31 |
| Decrease | Decrease | Increase | Increase | - | - | - | - | 102 | 32 | - | - |
| Decrease | Decrease | Increase | Decrease | 164 | 56 | 16 | 40 | 55 | 2,607 | 5,243 | 7,371 |
| Decrease | Increase | Decrease | Increase | - | - | - | - | 5,663 | - | - | 304 |
| Decrease | Increase | Decrease | Decrease | 35 | - | - | - | 662 | 5,475 | - | 42 |
| Decrease | Stable | Decrease | Increase | 9 | - | - | - | 38 | 361 | 7 | - |
| Decrease | Stable | Decrease | Decrease | - | - | - | - | - | - | - | - |
| Decrease | Decrease | Decrease | Increase | 121 | 628 | 822 | 8,144 | 436 | 463 | 99 | 493 |
| Decrease | Decrease | Decrease | Decrease | 145 | 34 | 2,887 | 200 | 2,802 | - | 19 | 887 |

LSF A37

| Capital | Number of vessels | Average value per vessel | NVA | 2009 | 2010 | 2011 | 2012 | 2013 | 2014 | 2015 | 2016 |
| --- | --- | --- | --- | --- | --- | --- | --- | --- | --- | --- | --- |
| Increase | Increase | Increase | Increase | 83 | 1,530 | 74 | 73 | 177 | 1,719 | 1,424 | - |
| Increase | Increase | Increase | Decrease | 111 | 1,142 | 158 | 214 | 571 | 244 | 59 | 2,170 |
| Increase | Stable | Increase | Increase | - | - | 28 | - | - | 7 | - | 69 |
| Increase | Stable | Increase | Decrease | - | - | 145 | - | 13 | 12 | 17 | 47 |
| Increase | Increase | Decrease | Increase | 14 | - | - | 1,961 | 1,222 | 1,086 | - | 17 |
| Increase | Increase | Decrease | Decrease | 564 | 501 | 1,797 | 544 | - | 200 | 574 | 441 |
| Increase | Decrease | Increase | Increase | 3,224 | 1,025 | 13 | 1,953 | 4,432 | 104 | 1,407 | 227 |
| Increase | Decrease | Increase | Decrease | 509 | 1,275 | 637 | 658 | 533 | 259 | 1,971 | 987 |
| Decrease | Decrease | Increase | Increase | 1,317 | 53 | 648 | 1,733 | 2,366 | 472 | 1,676 | - |
| Decrease | Decrease | Increase | Decrease | 44 | 1,334 | 185 | - | 78 | 1,701 | 633 | 1,763 |
| Decrease | Increase | Decrease | Increase | 1,852 | 2,295 | 183 | - | 853 | 1,525 | 611 | 35 |
| Decrease | Increase | Decrease | Decrease | - | 1,712 | 4,093 | 2,444 | 15 | 1,317 | 1,851 | 4,178 |
| Decrease | Stable | Decrease | Increase | 527 | 124 | - | 73 | 12 | - | - | 83 |
| Decrease | Stable | Decrease | Decrease | - | 118 | 33 | 101 | - | - | 229 | - |
| Decrease | Decrease | Decrease | Increase | 4,028 | 1,796 | 3,455 | 684 | 2,208 | 1,134 | 382 | 1,288 |
| Decrease | Decrease | Decrease | Decrease | 1,434 | 687 | 1,508 | 2,493 | 429 | 3,217 | 1,886 | 1,472 |

SCF A27

| Capital | Number of vessels | Average value per vessel | NVA | 2009 | 2010 | 2011 | 2012 | 2013 | 2014 | 2015 | 2016 |
| --- | --- | --- | --- | --- | --- | --- | --- | --- | --- | --- | --- |
| Increase | Increase | Increase | Increase | 32 | 2,608 | 16 | 26 | 11 | 717 | 69 | 7 |
| Increase | Increase | Increase | Decrease | 485 | 9 | 13 | 158 | 31 | 154 | 734 | 134 |
| Increase | Stable | Increase | Increase | - | 716 | - | 18 | - | 43 | 733 | - |
| Increase | Stable | Increase | Decrease | - | 2 | 6 | 16 | 30 | 12 | 41 | 32 |
| Increase | Increase | Decrease | Increase | 93 | 222 | 8 | 251 | - | 632 | 7 | - |
| Increase | Increase | Decrease | Decrease | 148 | 59 | 29 | 42 | - | 39 | 69 | 86 |
| Increase | Decrease | Increase | Increase | 157 | 259 | 45 | - | 161 | 63 | 194 | 91 |
| Increase | Decrease | Increase | Decrease | 2,860 | 179 | 16 | 195 | 112 | 89 | 469 | 857 |
| Decrease | Decrease | Increase | Increase | 139 | 37 | 233 | 83 | 137 | 310 | 21 | 92 |
| Decrease | Decrease | Increase | Decrease | 50 | 12 | 96 | 190 | 6 | 42 | 188 | 36 |
| Decrease | Increase | Decrease | Increase | 21 | 102 | 1,388 | 21 | 34 | 25 | 28 | 504 |
| Decrease | Increase | Decrease | Decrease | 553 | 36 | 25 | 157 | 14 | 531 | 24 | 1,232 |
| Decrease | Stable | Decrease | Increase | 9 | 73 | 76 | 28 | 92 | 216 | 181 | 4 |
| Decrease | Stable | Decrease | Decrease | 50 | 18 | 26 | 26 | 73 | - | 63 | 35 |
| Decrease | Decrease | Decrease | Increase | 51 | 1,084 | 2,896 | 3,879 | 1,686 | 1,321 | 581 | 357 |
| Decrease | Decrease | Decrease | Decrease | 1,099 | 228 | 578 | 162 | 2,575 | 803 | 1,477 | 1,351 |

LSF A27

| Capital | Number of vessels | Average value per vessel | NVA | 2009 | 2010 | 2011 | 2012 | 2013 | 2014 | 2015 | 2016 |
| --- | --- | --- | --- | --- | --- | --- | --- | --- | --- | --- | --- |
| Increase | Increase | Increase | Increase | 525 | 99 | 149 | 55 | 362 | 418 | 259 | 83 |
| Increase | Increase | Increase | Decrease | 277 | 413 | 239 | 295 | 541 | 739 | 629 | 771 |
| Increase | Stable | Increase | Increase | 159 | 33 | 105 | 97 | 331 | 43 | 49 | 45 |
| Increase | Stable | Increase | Decrease | 72 | 129 | 107 | 106 | 134 | 70 | 130 | 66 |
| Increase | Increase | Decrease | Increase | 8 | 185 | 88 | 135 | 157 | 63 | 13 | - |
| Increase | Increase | Decrease | Decrease | 143 | 108 | 328 | 243 | 56 | 156 | 63 | 106 |
| Increase | Decrease | Increase | Increase | 664 | 181 | 405 | 404 | 825 | 230 | 563 | 129 |
| Increase | Decrease | Increase | Decrease | 147 | 635 | 75 | 646 | 195 | 782 | 570 | 541 |
| Decrease | Decrease | Increase | Increase | 175 | 650 | 252 | 451 | 107 | 113 | 504 | 288 |
| Decrease | Decrease | Increase | Decrease | 110 | 711 | 101 | 294 | 212 | 194 | 184 | 105 |
| Decrease | Increase | Decrease | Increase | 196 | 232 | 133 | 63 | 366 | 58 | 248 | 178 |
| Decrease | Increase | Decrease | Decrease | 576 | 105 | 668 | 295 | 193 | 270 | 422 | 342 |
| Decrease | Stable | Decrease | Increase | 132 | 236 | 107 | 111 | 217 | 135 | 75 | 107 |
| Decrease | Stable | Decrease | Decrease | 127 | 123 | 134 | 227 | 199 | 208 | 231 | 79 |
| Decrease | Decrease | Decrease | Increase | 1,263 | 999 | 997 | 864 | 870 | 609 | 345 | 309 |
| Decrease | Decrease | Decrease | Decrease | 1,317 | 722 | 1,513 | 886 | 300 | 917 | 625 | 1,586 |

Figure SM2.1: a) Share of fleets with increasing capital, with increasing NVA and showing positive NVA by sea basin and scale.


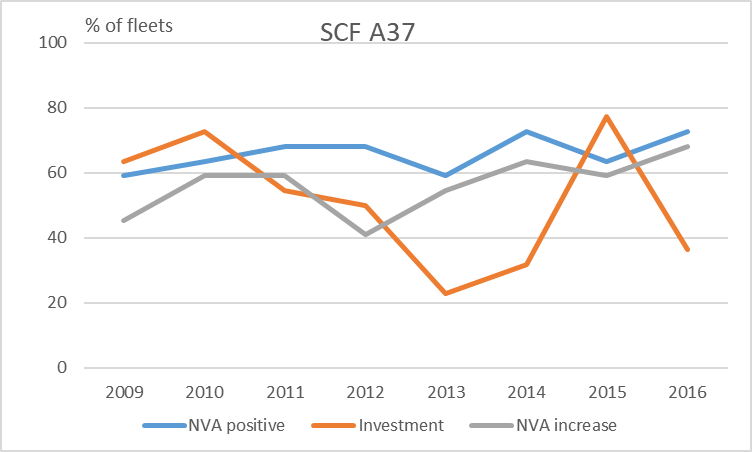

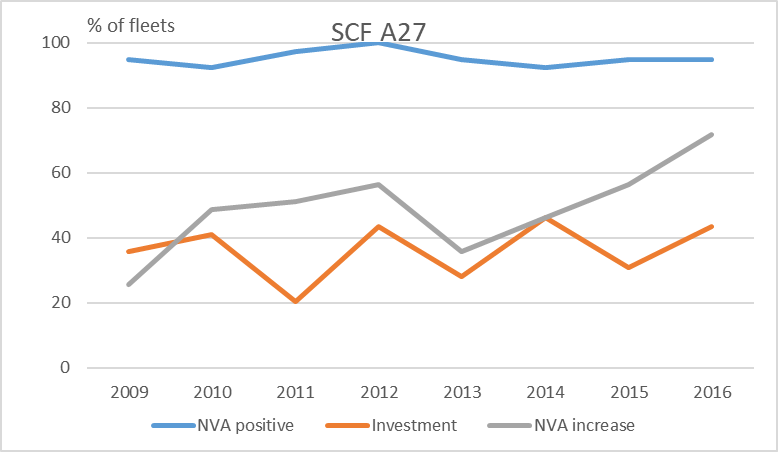


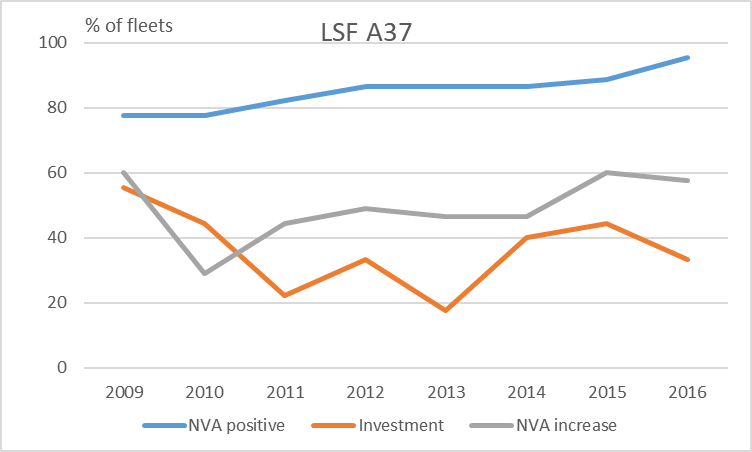

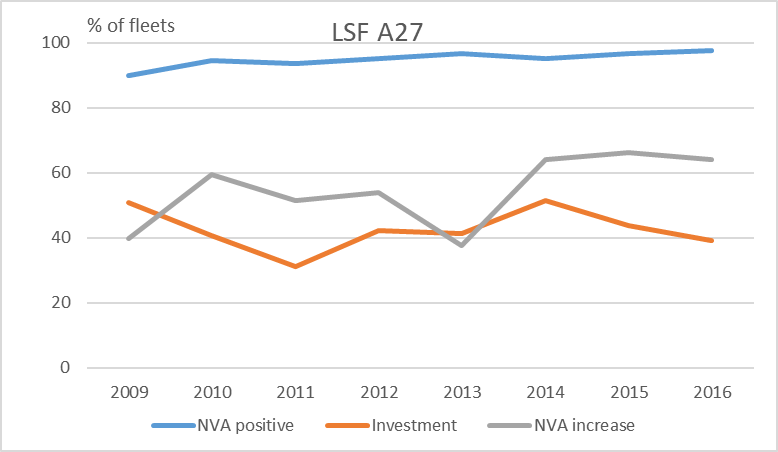


Figure SM2.1: b) Share of vessels by fleets with increasing capital, with increasing NVA and showing positive NVA by sea basin and scale.


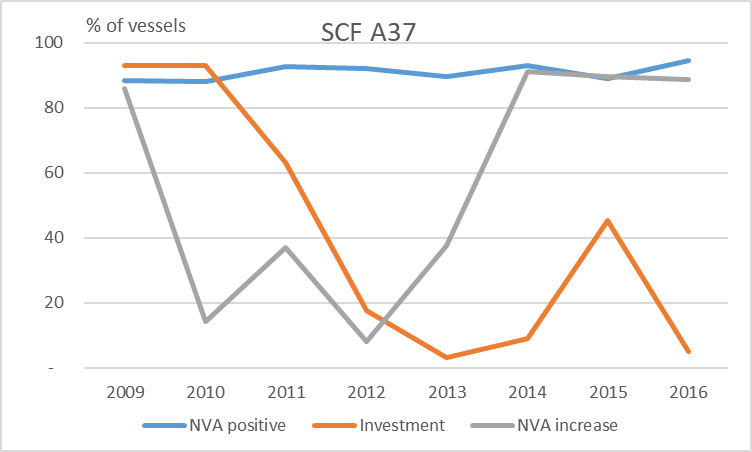

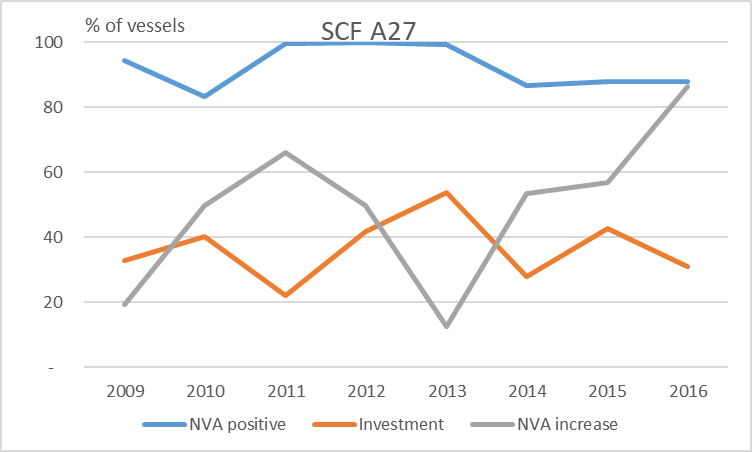


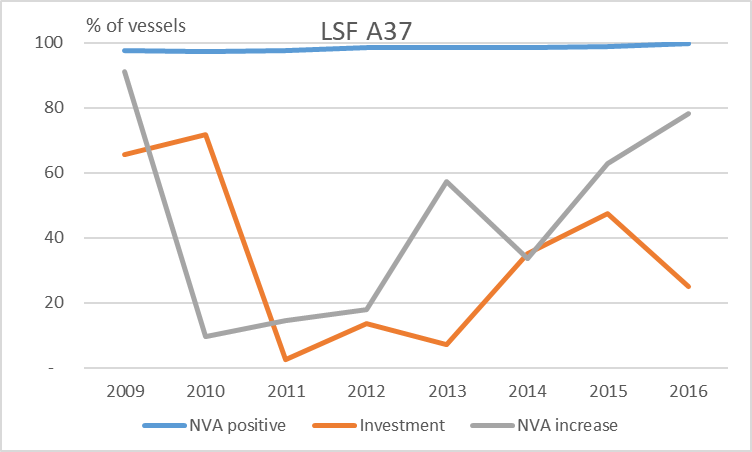

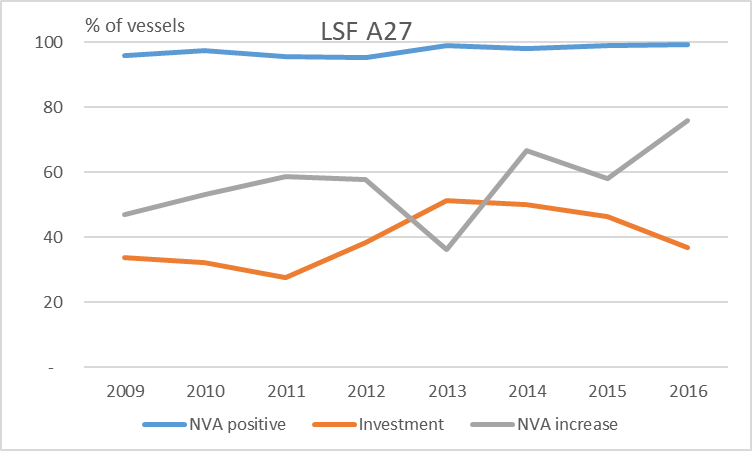


Figure SM2.2a: Evolution of investment types (in %) for the EU fleets analysed between 2008 and 2016.


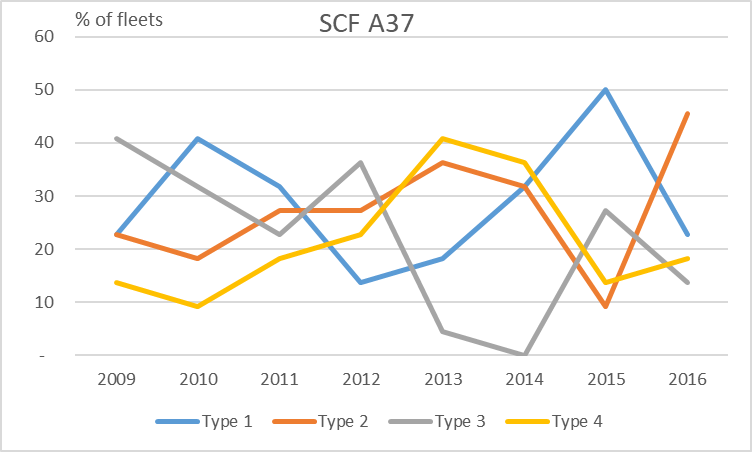

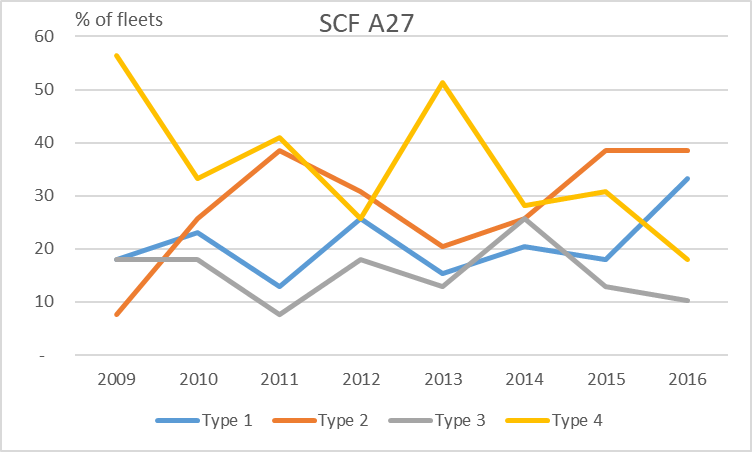


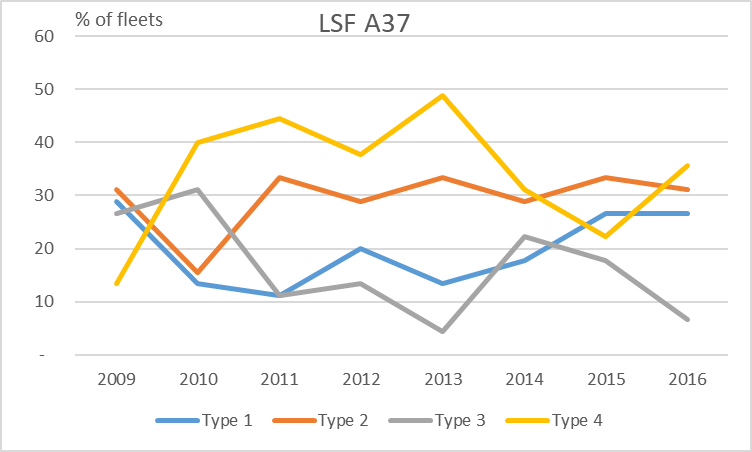

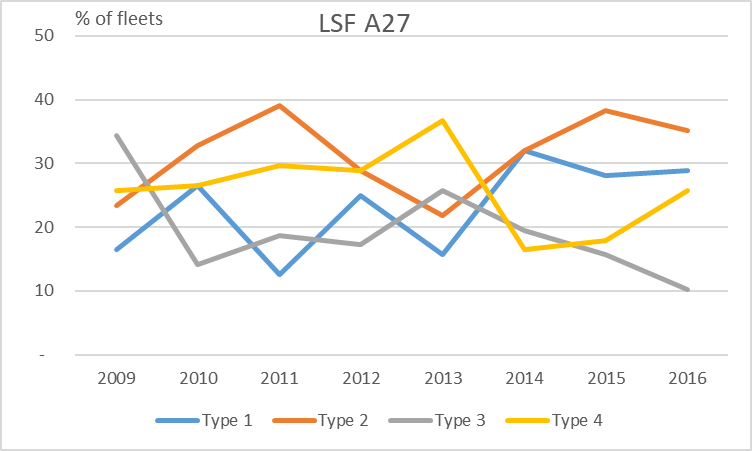


Figure SM2.2b: Evolution of investment types (in %) for the EU fleets by vessel analysed between 2008 and 2016.


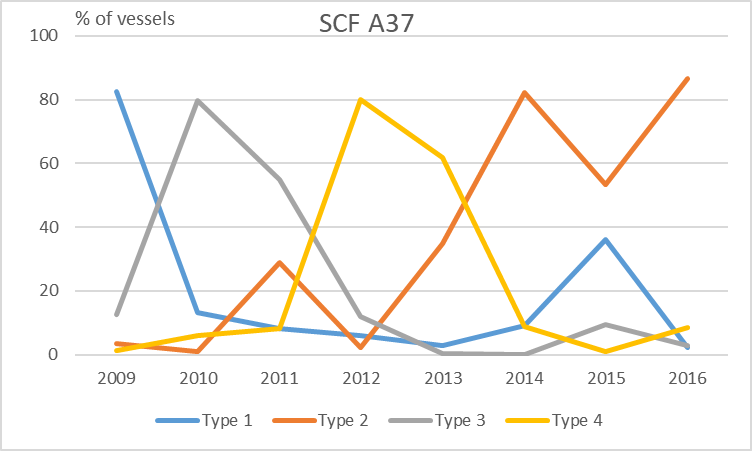

Supplement: Supplementary file 2 [file mmc2.docx]
