# Supplementary material for: Characterising investments in EU fisheries and defining their desirability
Source: Fish Res. 2020 Jan;221:105396. doi: 10.1016/j.fishres.2019.105396 (PMC6853412; doi:10.1016/j.fishres.2019.105396)
Supplement: Supplementary file 3 [file mmc3.docx]

**Appendix 1**

**1. Theoretical approach: a bio-economic model illustration**

In order to illustrate the capital and investment theories, we use a traditional static bio-economic model approach. The growth of a fish stock can be expressed by the continuous version of the logistic model described by the differential equation used in the Verhulst/Pearl surplus production model (eq. A.1; Schaefer, 1970). Changes in biomass of an exploited population can then be expressed as:

$\frac{dB}{dt}=r B \left(1-\frac{B}{K}\right)-H$ (A.1)

Where B is biomass, dB/dt is the temporal change in B, r is the intrinsic rate of natural population growth, K is the environmental carrying capacity for the population, and H is the biomass extracted in the form of catch (harvest).

The short-run harvest function follows the common Schaefer harvest function (eq. A.2; Schaefer, 1954), where the harvest is proportional to the fishing effort (i.e., inputs by time devoted to fishing) and the stock biomass. In the steady state (*dB/dt = 0*) the sustainable harvest rate is derived from equation A.3. Thus, harvest follows a parabolic curve as a function of fishing effort, with a maximum harvest called the Maximum Sustainable Yield (MSY).

$H=q\cdot E\cdot B$ (A.2)

$H=qKE-\frac{q^{2}K}{r}E^{2}$ (A.3)

Where q is the catchability coefficient and E is the fishing effort. The catchability coefficient (*q*) expresses how effective the fishing effort is in relation to the stock level. Usually q is assumed to be constant while E is variable.

The total revenues (*TR*) of a fishery in a given year (*t*) is equivalent to the value of landings (VL), and so equals the quantity harvested multiplied by the price of fish (*p*) (eq. A.4). Fishing costs (TC) are assumed linearly proportional to effort at a constant cost per unit of effort (c); in other words, it is assumed to reflect constant outlays to scale (eq. A.5). Thus, operating profit (Π) can therefore be estimated by the difference between the total revenues and the total costs (*TC*) (eq. A.6).

TR_t_ = VL_t_ = p H_t_ (A.4)

TC_t_ = c E_t_ (A.5)

Profits_t_ = Π_t_ =TR_t_ – TC_t_ (A.6)

These relationships are illustrated in Figures A.1a and A.1b. As increasing amounts of labour and capital inputs are used, total revenues from the fishery increases but with diminishing returns to scale. However, beyond a certain point, total revenues will decrease with increasing use of inputs (i.e., production factors) and a consequent increase in costs.


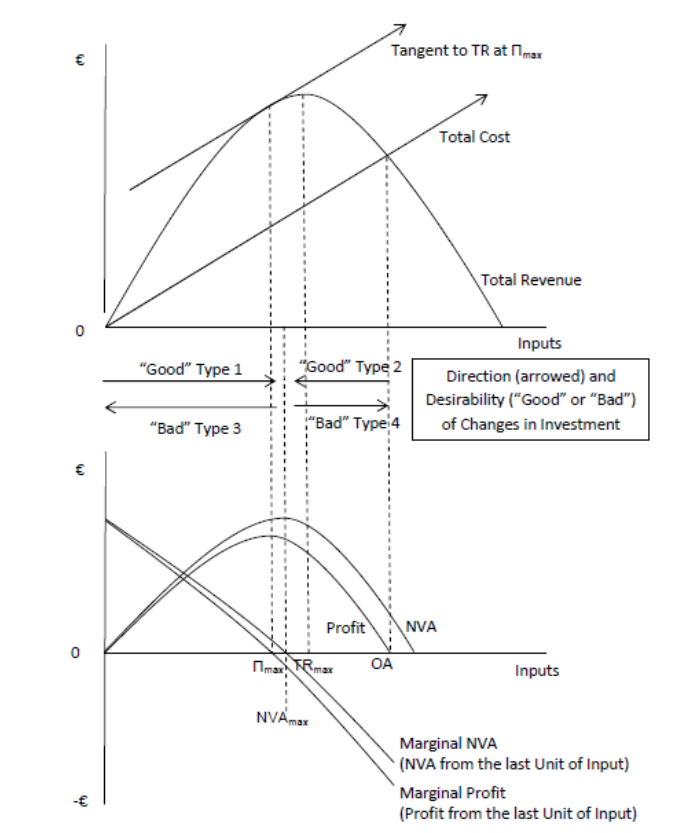


Figure A.1a,b: Representation of the relation between capitalisation and economic performance in fisheries and their impact on the desirability of investment

Bio-economic equilibrium in open access (OA)^[[1]](#footnote-1)^ fisheries occurs where TR equals TC. Fisheries, when there is absent or poor management, tend to this level because vessels can continue to obtain profits beyond the level that achieves the maximum profits and so inputs will tend to increase up to the point where profits dissipate at the bio-economic equilibrium. Any level of TR below the maximum, which with constant prices equates to the long-run equilibrium MSY, can be obtained at two levels of input. Obviously, the use of more inputs to achieve the same level of production is economically inefficient, because it incurs unnecessarily higher costs and is wasteful of input resources.

MEY corresponds to the level of inputs where maximum profits (Π_max_) can be obtained (i.e., where there is a maximum difference between the TR and TC). At Π_max,_ the gradient of the tangent to the TR curve equals that of the TC line. From the economic perspective, the use of inputs at levels greater than necessary to obtain MEY are inefficient, wasteful of input resources and are therefore undesirable. MEY is in most cases achieved with lower inputs than required to achieve MSY and at higher levels of stock size (stock capital). Hence, the point of maximum TR (TR_max_ or MSY) cannot be regarded as an economic optimal^[[2]](#footnote-2)^.

To determine whether increases or decreases in inputs are economically beneficial, information regarding the current performance of the fishery is required. Such information can be obtained from observed trends in profitability. We propose to use Net Value Added (NVA), the sum of the returns to both capital (i.e., net profit) and labour (i.e., salaries), as a measure of profitability as it is generally regarded as a better means of defining the returns to society than using profits alone (Chen et al., 2005; Guillen et al., 2015). In doing so, the desired reference point is shifted from maximum profitability to maximum NVA (NVA_max_), a position between MEY and MSY, but it does not affect the logic of the analysis previously detailed. Therefore, changes in the level of inputs (i.e., investments or disinvestments) towards NVA_max_ may be considered desirable, (“good” in Figure A.1a), because they represent a move towards maximum economic efficiency or, conversely, detrimental (“bad”) when they move away from NVA_max_.

Net value added is the value of output less the values of both intermediate consumption and consumption of fixed capital. Hence, we estimate NVA as:

NVA = TR – Fuel costs – Other variable costs – Repair and maintenance costs - Other non-variable costs – Depreciation costs (A.7)

Where capital is used as the measure of the amount of inputs used in the fishery. Capital is estimated as the tangible assets value, measured as the depreciated replacement value, as collected under the EU data collection framework (DCF). The variation of capital from year t-1 to year t can be considered as the real investment in capital (capital flow) taking place in a fleet.

Based on the above observations we can identify the basis for a decision rule to determine whether investments are, or have been, effective:

- When investments or disinvestments lead to increases in the NVA, they can be regarded as effective, heralding improved and sustainable long-run profitability of the fishery;
- When investments or disinvestments lead to decreases in the NVA, they can be regarded as detrimental to the long-run profitability of the fishery.

**References**

Chen, M.C., Cheng, S.J., Hwang, Y., 2005. An empirical investigation of the relationship between intellectual capital and firms’ market value and financial performance. J. Intellectual Capital, 6(2), 159-176. doi: 10.1108/14691930510592771.

Guillen, J., Calvo Santos, A., Carpenter, G., Carvalho, N., Casey, J., Lleonart, J., Maynou, F., Merino, G., Paulrud, A., 2016. Sustainability now or later? Estimating the benefits of pathways to Maximum Sustainable Yield for EU Northeast Atlantic Fisheries. Mar. Policy 72, 40-47. doi: 10.1016/j.marpol.2016.06.015.

Homans, F.R., Wilen, J.E., 1997. A Model of Regulated Open Access Resource Use. J. Environ. Econ. Manag. 32, 1-21. doi: 10.1006/jeem.1996.0947.

Schaefer, M.B., 1970. Men, birds and anchovies in the Peru current—dynamic interactions. Trans. Am. Fish. Soc. 99(3), 461-467. doi: 10.1577/1548-8659(1970)99<461:MBAAIT>2.0.CO;2.

Schaefer M.B., 1954. Some aspects of the dynamics of population important to the management of commercial marine fisheries. Bull. I-ATCC. 2, 247-85.

Sumaila, U. R., Hannesson, R., 2010. Maximum economic yield in crisis? Fish Fish. 11(4), 461-465. doi: 10.1111/j.1467-2979.2010.00381.x.

Wilen, J.E., 1985. Towards a theory of the regulated fishery. Mar. Resour. Econ. 1(4), 369-388. doi: 10.1086/mre.1.4.42628868.

1. Here “open access” is understood in its broader sense; in other words, it means all fisheries that are not bound by an economic cost that constrains effort. Distinction between “pure” open access and “regulated” open access is needed (Wilen, 1985; Homans and Wilen, 1997). A “pure” open access fishery is one in which the property rights are ill defined, or non-existent, and in which there is an absence of regulations governing the exploitation of the resource. While, a “regulated” open access fishery is a fishery with ill-defined property rights, in which the authorities rigidly control the global harvest in order to conserve the resource, but in which they do not exert effective control over vessel participation in the fishery. [↑](#footnote-ref-1)
2. See Sumaila and Hannesson (2010) for a discussion on the topic. [↑](#footnote-ref-2)
